# Factors to consider when designing post-hospital interventions to support critical illness recovery: Systematic review and qualitative evidence synthesis

**DOI:** 10.1177/17511437241308674

**Published:** 2025-01-03

**Authors:** Jonathan Stewart, Ellen Pauley, Danielle Wilson, Judy Bradley, Nigel Hart, Danny McAuley

**Affiliations:** Queen’s University Belfast, Belfast, UK

**Keywords:** Critical illness, transitions of care, hospital discharge

## Abstract

**Background:**

Survivors of intensive care unit (ICU) admission experience significant deficits in health-related quality of life due to long-term physical, psychological, and cognitive sequelae of critical illness, which may persist for many years. There has been a proliferation of post-hospital interventions in recent years which aim to support ICU-survivors, however there is currently limited evidence to inform optimal approach. We therefore aimed to synthesise factors which impacted the implementation of these interventions from the perspective of healthcare providers, patients, and their carers, and to compare different intervention designs.

**Methods:**

We conducted a systematic review and synthesis of qualitative evidence using four databases (MEDLINE, EMBASE, CINAHL and Web of Science) which were searched from inception to May 2024. The extraction and synthesis of factors which impacted intervention implementation was informed by the domains of the Consolidated Framework for Implementation Research (CFIR) and Template for Intervention Description and Replication (TIDieR) checklist.

**Results:**

Thirty-seven studies were included, reporting on a range of interventions including follow-up clinics and rehabilitation programmes. We identified some overarching principles and specific intervention component and design factors which may support in the design of future strategies to improve outcomes for ICU survivors. For each intervention characteristic, various patient, staff, and setting factors were found to impact implementation. Considering how the intervention will rely on and integrate with existing outpatient and community resources is likely to be important.

**Conclusion:**

This review provides a framework to future research examining the optimal approach to supporting ICU survivor recovery following hospital discharge.

## Background

Advances in intensive care unit (ICU) care have led to an increasing proportion of patients surviving critical illnesses which would have been almost universally fatal in the past.^1^ While these advances are welcome, much less research has been done to understand the optimal approach to support these ICU survivors after hospital discharge. Patients commonly experience long-term physical, psychological, cognitive sequalae which can persist for many years after ICU discharge,^2^ and lead to significant impacts on quality of life and potentially preventable hospital readmission.^3^ A significant proportion of previous research has focused on inpatient physical rehabilitation programmes, often implemented within the ICU.^4^ The evidence of benefit for these inpatient interventions remains inconclusive.^5,6^ Recently, there has been a proliferation of post-hospital interventions in clinical practice and research, including ICU follow-up clinics.^7,8^ Despite their widespread implementation, previous quantitative systematic reviews have demonstrated there is currently limited prospective evidence on the optimal approach to support patient recovery.^6,9,10^

In the absence of quantitative evidence to inform the optimal approach to support patient recovery after hospital discharge, to aid in the development of future interventions a lot can be learned from by synthesising qualitative data from previous studies related to the experiences of intervention implementation.^10^ This study aimed to synthesise factors which impacted the implementation of post-hospital interventions to support patient recovery after critical illness from the perspective of patients, carers and healthcare providers, and to compare different design options.

## Methods

### Study design

This systematic review and qualitative evidence synthesis was informed by Cochrane guidance.^11,12^ Reporting was informed by enhancing transparency in reporting the synthesis of qualitative research (ENTREQ) guidance.^13^ The systematic review was registered on the international Prospective Register of Systematic Reviews (PROSPERO; CRD42022364005).

### Criteria for considering studies for this review

The eligibility criteria for the studies included are summarised in Table 1.

**Table 1.** Study inclusion and exclusion criteria.

Study characteristics	Inclusion	Exclusion
Study designs	• Qualitative • Mixed methods	• Quantitative • Protocol • Review
Participants	Qualitative data from the perspective of; • Adults (⩾18 years) previously admitted to ICU; or • Carer of patients previously admitted to ICU; or • Healthcare professionals providing care to patients following hospital discharge after ICU admission	
Population of interest	• Adults (⩾18 years) previously admitted to an Intensive Care Unit	• Children (<18 years) • Subpopulations (neurological ICU, Cardiac ICU) • Psychiatric ICU
Setting(s)/Timing	• At or after hospital discharge after ICU admission.	
Intervention	• Interventions, programmes, or strategies to support patient recovery following critical illness	• End of life care, palliative care, bereavement interventions
Comparator	• Nil	
Outcomes	• Barriers and facilitators to implementation	
Publication	• Studies published in English • Publication from inception to present	• Non-English-language studies • Grey literature • Conference abstracts • Protocols

*Types of studies*: We included primary qualitative studies which used qualitative methods for data collection and analysis. We also included primary studies that use a mixed methods study design where it is possible to extract relevant qualitative data. We only included full text peer-reviewed articles published in English. We did not exclude studies based on our assessment of methodological limitations; however, we used this information to assess our confidence in the review findings. The reference lists of relevant review articles were screened for potential articles for inclusion.

*Setting*: Studies were included if an intervention was delivered at or after hospital discharge.

*Population*: Studies were included where data was available from the perspective of;

- Adults (⩾18 years) previously admitted to ICU; or
- Carers or relatives of patients previously admitted to ICU; or
- Healthcare professionals providing care to patients who were previously admitted to ICU.

*Intervention*: We included studies of interventions to support patient recovery following critical illness, including multicomponent interventions. We excluded end-of-life care, palliative care or bereavement interventions. We also excluded interventions focused exclusively on the needs of carers or relatives.

*Outcomes*: We included studies which provided data on factors which impacted the implementation of interventions.

### Selection of studies

**Figure 1.**
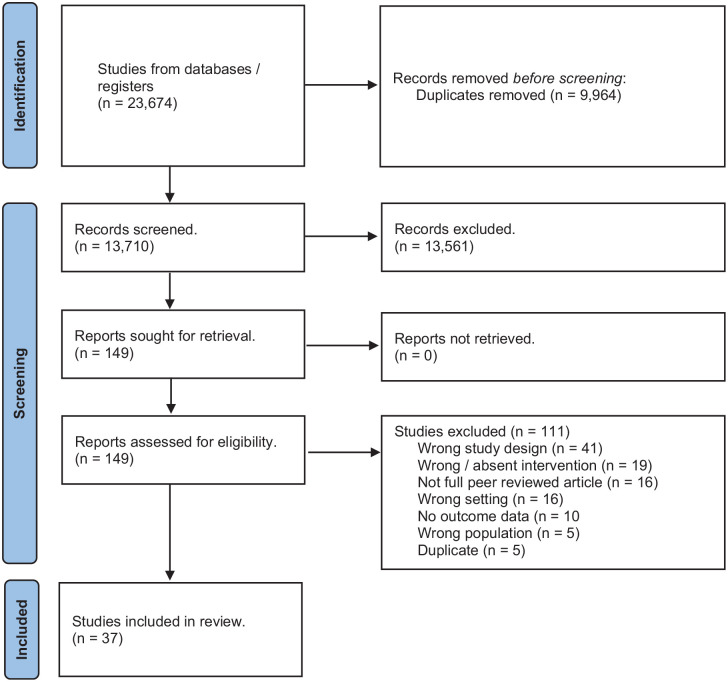
PRISMA flow diagram of study selection process. Source: Page MJ, McKenzie JE, Bossuyt PM, Boutron I, Hoffmann TC, Mulrow CD, et al. The PRISMA 2020 statement: an updated guideline for reporting systematic reviews. BMJ 2021;372:n71. doi: 10.1136/bmj.n71.

Four databases were searched (MEDLINE, EMBASE, CINAHL and Web of Science), along with the reference lists, from inception until 23rd May 2024 (Supplemental Appendix 1). Searches were validated by a university subject librarian. Covidence systematic review software (Veritas Health Innovation, Melbourne, Australia) was used to assist article screening. Two review authors independently assessed articles for inclusion. Disagreements were resolved by discussion or, when required, by involving a third review author. A PRISMA flow diagram is included to show our search results and the process of screening and selecting studies for inclusion (Figure 1).

### Choice of theory to guide data extraction and synthesis

Several theories were considered to inform data extraction and synthesis. The Consolidated Framework for Implementation Research (CFIR) was selected based on its ability to manage both breadth and depth of data for capturing the complexity of implementation of included interventions.^14^ The CFIR framework divides implementation into five major domains; (1) intervention characteristics, (2) inner setting (factors related to the setting where intervention was delivered), (3) outer setting (factors outside the setting where the intervention was delivered), (4) characteristics of the individuals involved in the implementation process and/or who use the intervention (healthcare professionals and patients), and (5) process of implementation. CFIR has been successfully used in a previous primary qualitative study which examined factors which impact the implementation of two types of interventions to support people following critical illness (follow-up clinics and peer support groups).^15^

### Data extraction

Data was extracted independently by two authors in two stages. In the first stage, data was extracted using Covidence software on each included study including descriptive characteristics, design, objectives, population, and reported findings. Data on each intervention was extracted using a customised data extraction framework, informed by the template for intervention description and replication (TIDieR) checklist.^16^

In the second stage, factors which impacted intervention implementation were coded using NVivo software (Lumivero NVivo Version 13). Two authors coded a sample (10%) of articles using an initial coding framework informed by the domains of the Consolidated Framework for Implementation Research (CFIR).^14^ Coding discrepancies were discussed, and the coding framework was updated. The final ‘best-fit’ framework was then be applied to all remaining articles.

### Methodological limitations of included studies

Two review authors independently assessed the methodological limitations of each study using the critical appraisal skills programme (CASP) tool.^17,18^ Disagreements were resolved by discussion or, when required, by involving a third review author.

### Data management, analysis and synthesis

A ‘best-fit’ framework synthesis approach was used for data analysis and synthesis, combining deductive framework synthesis and inductive thematic synthesis approaches.^19–21^ Primary qualitative findings were extracted and synthesised according to CFIR informed ‘best fit’ framework. Findings were then arranged according to the domains of the TIDieR checklist.

### Assessing confidence in the review findings

The GRADE-CERQual (Confidence in the Evidence from Reviews of Qualitative research) approach was used to assess confidence in each finding based on four components^17,22^: methodological limitations of included studies, coherence of the review finding, adequacy of the data contributing to a review finding and relevance of the included studies to the review question. After assessing each of the four components, a judgement was made about the overall confidence in the evidence supporting the review finding (high, moderate, low or very low). Final assessment was based on consensus among the review authors.

### Review author reflexivity

The authors are a multidisciplinary group of researchers and clinicians focused on support and rehabilitation for people following critical illness. They have and are currently engaged in several research studies on the development of and testing interventions to support people following critical illness. Given this current and prior experience, some biases may exist regarding preconceived ideas on factors which impact implementation of interventions following critical illness.

## Results

### Search results

The search generated 13,710 unique citations (Figure 1). 149 articles were retrieved as potentially relevant, of which 37 met the inclusion criteria.

### Characteristics of included studies

Characteristics of included studies and interventions are reported in Table 2, Supplemental Appendix 2, and Supplemental Appendix 3. Most studies were conducted in the United Kingdom (UK), with smaller numbers of studies conducted in Sweden, United States of America (USA), Australia, Denmark, Germany, Netherlands, Ireland, Belgium and China. Most studies reported on follow-up clinics, programmes or recovery services, with remainder examining physiotherapy, primary care information sharing, follow-up visits to the ICU, psychological therapies, and peer-led support interventions. Qualitative data from the perspective of patients, caregivers, ICU staff, primary care staff and other healthcare staff was collected via a variety of methods including interviews, focus groups, open ended survey questions and participant observations.

**Table 2.** Summary of included studies.

Study characteristics	Characteristics	*n* = 37
Country of origin	UK	15
	Sweden	5
	USA	5
	Australia	4
	Denmark	3
	Germany	3
	Netherlands	2
	Ireland	1
	Belgium	1
	China	1
Intervention	Follow-up clinic/programme/recovery service	20
	Physiotherapy/physical rehabilitation	8
	Primary care information sharing/discharge summaries	7
	Peer led support	5
	Follow-up visit to the ICU	3
	Psychological therapy/rehabilitation	2
	Mobile app	1
	Screening tool	1
Design	Primary qualitative	22
	Mixed methods	15
Participants	Patients	22
	Caregivers	9
	ICU staff	15
	Primary care staff	7
	Other healthcare staff	4
Qualitative data collection	Interviews	29
	Focus groups	6
	Open ended survey questions	6
	Observations	4
	Notebook	1
	Learning sessions	1
Qualitative data analysis	Content analysis	14
	Thematic analysis	11
	Grounded theory	2
	Framework analysis	2
	Focused ethnography	1
	Other	4
	Unclear	3

### Quality appraisal of included studies

The overall quality of included studies was good (Supplemental Appendix 4). The most common quality domains where insufficient information was available to make a judgement were: the appropriateness of research design to address the aims of the research, relationship between the researcher and participants, and rigour of data analysis.

### Synthesis

**Figure 2.**
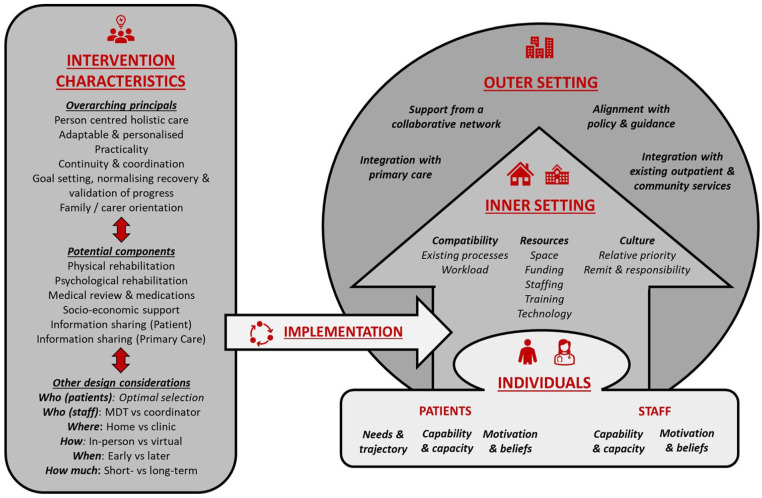
Factors which impact the implementation of post-hospital interventions to support ICU recovery. Potential factors are presented under the domains of the Consolidated Framework for Implementation Research (CFIR) and the Template for Intervention Description and Replication (TIDieR) checklist. For each intervention characteristic, various patient, staff, and setting factors were found to impact implementation. Source: Adapted from Damschroder et al.^14^

The objective of this study was to synthesise factors which impacted the implementation of post-hospital interventions to support ICU recovery. Extraction and synthesis of potential factors was informed by CFIR and TiDIER domains (Figure 2). We identified some overarching principles and specific intervention component and design factors which may support in the design of future strategies to improve outcomes for critical illness survivors. For each intervention characteristic, various patient, staff, and setting factors were found to impact implementation.

#### Overarching principals

Regardless of the type of intervention components, several overarching principals appear to be important when designing an intervention to support patient recovery after critical illness.

*Person centred holistic care*: ICU survivors have unique combinations of unmet functional, medical, and social issues following hospital discharge.^23–27^ Interventions should aim to comprehensively identify and address each patient’s specific unmet needs.^15,25,28–30^ Staff should aim to build rapport and trust with patients through effective communication.^15,25,28,31–34^

*Adaptable & personalised*: Interventions need be adaptable to each patients’ specific needs. The capability and capacity of patients to engage in any intervention is impacted by a range of factors,^27,29,30,32,35–41^ including their evolving functional status (e.g. physical and psychological),^27,29,30,32,35–41^ comorbidities,^26,32^ and health literacy.^42^ Their motivation to engage in ICU follow-up will be impacted by whether they have a clear understanding of its purpose and potential impact.^15,24,25,31,32,39,41^

*Practicality*: Intervention designers need to consider the practicality of an intervention for staff and patients.^24,30,42^ For example, while it may be preferable to deliver the intervention in-person in the patient’s home, this may not be practical due to factors such geographic distance from the admitting hospital.^15,24^

*Continuity and coordination of care*: ICU survivors’ transition between numerous care settings, including the ICU, acute hospital wards and primary care.^27,35,43,44^ This can lead to fragmented care and a lack of clarity on which of the patient’s various healthcare providers is responsible for follow-up after hospital discharge.^23,27,44,45^ A key role of ICU recovery services should be coordination of care.^23,29,34,37,43,45–47^ The interface with primary care was identified as particularly important, including the provision of adequate information by hospital staff to primary care providers to enable ongoing continuity of care.^26,27,44,45,48^

*Goal setting, normalising recovery & validation of progress*: Interventions should set personalised achievable goals and validate patient progress and recovery.^33,35,37,39,40,46,47^

*Involvement of informal carers*: Given the multiple sequalae of critical illness, family members and other informal carers often play important roles supporting patients in the post-hospital period, and consideration should be given on how best to involve and support them.^15,27,31,38–40,49,50^

#### Potential intervention components

While certain factors are relevant to any intervention to support recovery after hospital discharge, certain factors particularly impact specific potential components.

*Physical Rehabilitation*: Physical exercise approaches need to be accessible and adaptable to account for heterogenous patient needs.^32,35,42^ A number of strategies were identified which may enhance patient motivation to engage with physical rehabilitation, including exercise supervision^32^ or a group based programme.^35^ Consideration needs to be given to how the intervention will link to other existing community or outpatient rehabilitation services to avoid duplication and optimise use of resources.^8,15,24,35,42,44,45,51^

*Psychological rehabilitation (psychological therapy, information provision, ICU visits & peer support)*: Potential strategies to support psychological recovery include psychological therapy, information sharing, an ICU visit and peer support. Psychological therapy needs to be integrated with other forms of support.^30,34,40^ Patients will vary in terms of their capability and capacity to return to the admitting hospital for therapy (e.g. physical functional or triggering effect of the hospital).^30,40^ Provision of information may support normalisation and expectation management to help patients make sense of their ICU experience and recovery.^23,27,30,31,37^ Incorporating a return visit to the ICU setting enables patients to meet the staff who cared for them and see the ICU room.^28,31,46,52,53^ A number of adjuncts were identified which could support an ICU visit, including photographs taken when critically ill^52,54^ and review of an ICU diary.^46,52–54^ However, the patient needs to be capable of travelling to the hospital, which they may not be willing or able to.^53^ Peer support groups may also help patients make sense of their ICU experience and recovery, by meeting other people who have been through a similar experience.^15,36,40^ There are several practical considerations including whether the discussion should be facilitated and whether family and carers should be present.^15,36,55^

*Medical review (including medicine optimisation)*: Patients often have medical issues which require follow-up after hospital discharge, including new and existing medical conditions and medications.^37,46^ Staff engagement is dependent on their clarity on the purpose of incorporating a medical review (e.g. comprehensive screening for unmet needs, follow-up of tests and referrals, or medication review).^29,37,46^ Furthermore, incorporating medical review requires access to staff with the required experience.^8,29,34,40^

*Socio-economic support*: There is increasing recognition of the social and economic sequalae of critical illness, such as social isolation and employment disruption.^38,46^ Incorporating support for these issues into an ICU recovery programme requires access to the required expertise which may vary by locality.^38,46^ This may be supported by strengthening links with existing social and community services.^38,46^

*Information sharing with primary care*: The ability of the patient’s primary care provider to provide ongoing continuity and coordination of care will be dependent on the quality and timeliness of the information they receive.^26,27,44,45,47,48,50^ This information is often delayed,^27,44^ and provided by hospital ward staff who may lack awareness of ICU sequalae.^27,44,47,50^ The ability of primary care teams to act on the information received may be inhibited if they have limited experience of potential ICU sequalae,^23,26,27,37,47^ a lack of clarity on their remit and responsibility following hospital discharge,^27,44,45,47^ and lack of capacity due to other workload pressures.^26,27,47^

#### Other design considerations

In addition to deciding what components to include in an intervention, there are a variety of other design characteristics which need to be considered.

*Who (Patients)*: Given the heterogeneity of patients following ICU, several included articles highlighted the importance of optimal patient selection, to ensure interventions target patients most likely to benefit within resource constrained healthcare systems.^15,23,42^ However, they were unable to agree on precise criteria, highlighting the need for future work in this area.

*Who (Staff)*: The staff who deliver the intervention need to have the required knowledge and experience of potential critical illness complications, which may include awareness of problems outside their professional scope.^8,15,23,25,26,28,31–34,40–42,50,56^ Multicomponent interventions which aim to comprehensively address unmet functional, medical, and social issues may require staff from a variety of professional backgrounds.^8,15,23,42^ The need for large complex multidisciplinary team may limit the scalability of an intervention, particularly given the current staffing, funding and workload resource challenges across health systems.^7,8,15,23,24,38,42,43,45,47,57^ An alternative strategy adopted by some included studies was utilisation of a care coordinator who identifies problems and coordinates with other professional groups where required.^25,26,28,31,33^ This may be a more efficient use of resources and enable staff to develop a therapeutic relationship with patients over time.^15^ However, the problems which can be identified and addressed are dependent on the skills and experience of the staff member^25,32^ and their ability to access existing outpatient and community services when required.^8,15,24,35,42,44,45^

*Where (Home* vs *clinic)*: The chosen setting for the intervention needs to be adequately supportive and resourced, with the required funding,^7,15,23,38,42,43^ staffing^7,8,43,45^ and space.^15,38,43^ Hospital based follow-up clinics enable multiple professionals to be present within a single clinic, potentially reducing treatment burden for the patient and increasing convenience of delivery for staff.^7,15,33,37,38,43,46,49,57^ However some patients may be inhibited from returning to the hospital by physical and psychological complications of critical illness^25,26,32,33,37^ or geographic distance.^15,24,25^ Delivery at home has the potential to enhance access and reduce attrition by facilitating early support where functional impairment or inability to drive impedes hospital attendance.^25,42^ It may also be less resource intensive by integrating with existing outpatient and community services rather than duplication of resources, however this relies on access to these services (e.g. physical or psychological rehabilitation) which is variable.^8,15,24,35,42,44,45,51^ A potential compromise could be a hybrid approach, which blends clinic and home based care.^35^

*How (In-person* vs *virtual)*: Some staff in included studies felt virtual care was convenient and becoming normalised as part of routine care.^24,28,41,58^ However, certain intervention components require significant adaptation for virtual delivery such as physical rehabilitation and ICU ward visits.^28,40–42^ Virtual care may enhance access for patients with significant sequalae from their illness which could make travel challenging,^25,26,32,33,37^ and for those who live a long distance from the admitting hospital.^15,24,25^ However, it also has the potential to exacerbate existing digital inequalities for patient who lack access or ability to use the required technology, or with hearing or visual impairment.^24,58^

*When (Early* vs *later)*: The timing of an intervention will be dependent on compatibility with specific intervention components^31,38^ and the variable recovery trajectories of patients.^8,23,25,28,30,31,35,37,39,42,53^ Interventions need to start early enough in the recovery period to identify and address potential issues prior to deterioration,^8,31,35,37^ however long enough after ICU discharge for patients to be capable of engaging with an intervention.^25,28^

*How much (Short-* vs *long-term)*: The duration of the any programme will likely need to be flexible, to account for variable patient recovery trajectories.^25,31,34,37,40,42,45,56^ Some studies argued for longitudinal follow-up,^23,46^ however others recognised the need to balance supporting patients against development of dependency.^23,32,46,49,56^

### Assessing confidence in the review findings

The GRADE CERQual assessment was applied to the main review findings (Table 3 and Supplemental Appendix 5).^17,22^ Findings were categorised as high (28 findings), moderate (15 findings) or low (1 finding) confidence. The finding related to when the intervention is delivered and its compatibility with specific intervention components was rated as low due to moderate concerns about methodological limitation and adequacy (data from only two studies).

**Table 3.** GRADE-Confidence in the Evidence from Reviews of Qualitaitve Research (CerQual) assessment of study findings.

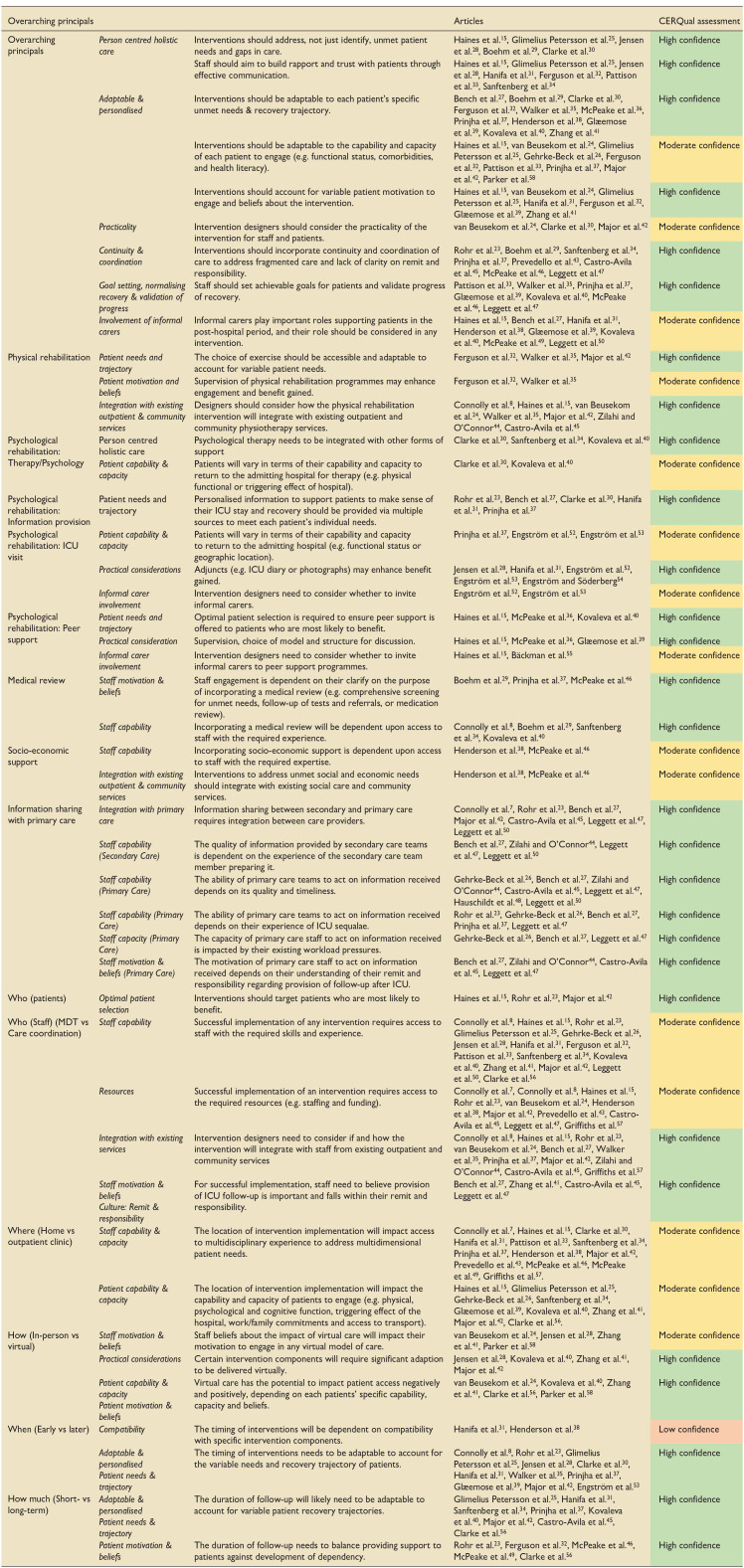

## Discussion

Despite the widespread implementation of various of post-hospital ICU interventions, there is currently a lack of evidence unpinning the optimal approach to support patient recovery.^6,9,10^ This review aimed to synthesise the experiences of patients, carers and healthcare professionals about implementation of post-hospital interventions, to inform the design of future interventions. A variety of potential intervention components were included which aimed to address unmet patient needs after ICU. Each component has their own set of design considerations including who will deliver it, where, when and for how long. Each potential component and design choice will be impacted by factors relating to the patients receiving the intervention, the staff delivering it and the setting where its delivered. By examining factors which impact multiple intervention types following hospital discharge, we demonstrate the additional complexity of bundling multiple components together into a multicomponent intervention. Factors which impact individual components and design options interact and may not be compatible with each other. It is therefore not surprising that it has been extremely challenging to create a one-size-fits-all model to address the unmet needs of this heterogeneous patient group. This may also help explain why specific chosen quantitative outcomes have failed to identify intervention benefit in previous clinical trials.^6,9,10^

Until relatively recently the focus of most research into how best to support patient recovery after critical illness had been on potential physical rehabilitation strategies to address ICU acquired muscle weakness, the majority of which were delivered prior to hospital discharge.^5,6,9^ A previous mixed methods systematic review outlined potential factors impacting the ability of ICU survivors to participate in physical rehabilitation interventions during and after critical illness.^4^ However, this study didn’t examine interventions to address other unmet needs after critical illness (e.g. psychological, social or medical), and only 7% of included studies assessed post-ICU interventions.

In the last two decades there has been a proliferation of different types of post-hospital interventions in clinical practice and research which aim to enhance ICU recovery.^7,8,57^ This widespread implementation has taken place despite quantitative systematic reviews identifying a lack of evidence for the optimum approach to enhance patient outcomes.^6,9,10^ However, syntheses of purely quantitative evidence don’t provide information on factors which impacted intervention implementation which may help explain why the interventions didn’t improve outcomes. By synthesising qualitative data on the experiences of patients, carers and healthcare professionals on intervention implementation, this systematic review providers a richer picture to help clarify the quantitative evaluations and enable comparison of potential intervention components and design options to inform the design of future interventions.

Based on the findings we make several recommendations for future studies. First, to account for the heterogeneous needs of critical illness survivors and likely need for complex multicomponent interventions to meet these needs, we provide some overarching principles as a framework to support future intervention design. Future interventions should include holistic assessment (biological, psychological and social) and likely need to be adaptable to account for individual patient unmet needs. The intervention needs to consider other important stakeholders including the role of informal caregivers, although interventions should be feasible in the absence of this support. This is consistent with previous studies from other settings which examined factors associated with delivery of high-quality care for patients with complex needs (e.g. frail elderly) as they transition from hospital to home.^59,60^

**Figure 3.**
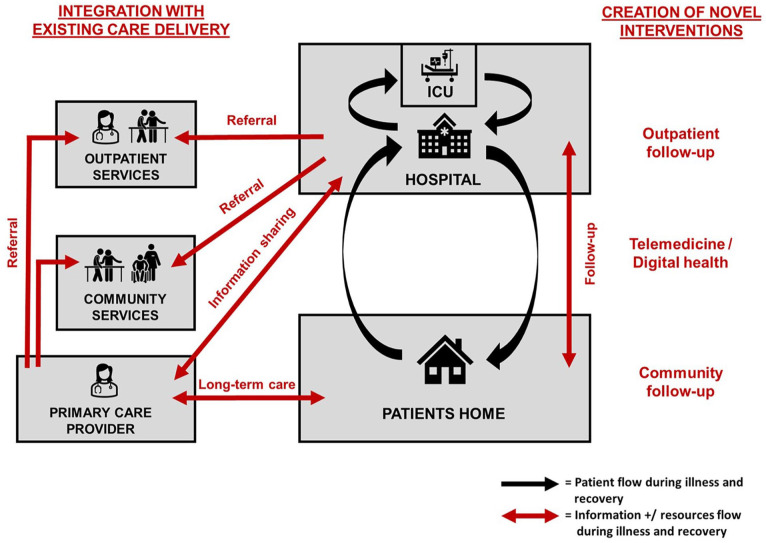
Create versus Integrate: A fundamental question for any novel post-hospital intervention to support patient recovery after critical illness is the degree to which it will rely on and integrate with existing outpatient and community resources, including the patients primary care provider.

Second, intervention designers need to carefully consider the degree to which any future intervention will rely on and integrate with existing outpatient and community resources, including the patients primary care provider (Figure 3). Any new intervention is likely to overlap with and potentially duplicate existing care provision and pathways. Many of the included interventions fell somewhere between the two extremes of a novel multidisciplinary follow-up clinic which relies entirely on new resources (including staff), and a single coordinator of care who integrates with existing resources when required. Both approaches have benefits and limitations, including ability to provide comprehensive holistic patient care, resources required and efficiency and combability and scalability within current healthcare systems. Regardless of the degree to which interventions intervention with existing outpatient and community resources, intervention designers need to consider how any future intervention will integrate with the patient’s primary care provider, to support ongoing continuity and coordination of care.

Third, any future studies which design and evaluate interventions aiming to support patients after critical illness should utilise mixed methods approaches which integrates qualitative and quantitative data.^61^ Given the heterogeneity of critical illness survivors, relaying on quantitative data is unlikely to provide a comprehensive evaluation of interventions and will likely miss important potential learning for future clinical practice and research.

Fourth, a significant current gap in the evidence base for interventions to support patient recovery after critical illness is an understanding of the health economics. Several included studies highlighted challenges of limited available resources and integrating interventions within already financially stretched public healthcare systems. It would be important to demonstrate the economic benefits of any proposed complex interventions at the patient (micro), healthcare systems (meso) and wider societal (macro) levels.

This study has several strengths and limitations. The study design was informed by guidance from Cochrane guidance.^11,12^ We incorporated duplicate article screening and data extraction to enhance review rigour. The inclusion criteria for the study were restricted to interventions delivered at the point of and after hospital discharge, as a key period in the recovery trajectory of patients following a critical illness. However, we recognise this needs to be considered as part of the whole patient journey, including events prior to and during the hospitalisation. While there may be subjectivity in the extraction and synthesis of the qualitative data, the generalisability of findings is enhanced by dual coding and synthesis, and the synthesis of qualitative data from multiple sources. While methodological limitations of included studies were dual assessed using the critical appraisal skills programme (CASP) tool,^18^ the study findings should be interpreted with caution as all studies were included regardless of this assessment. However, the clarity and robustness of the findings is enhanced by our evaluation of the certainty of evidence using the GRADECerQual tool (Table 3 and Supplemental Appendix 5). Data collection and analysis was informed by the domains of CFIR and TIDIER.^14,16^ CFIR was chosen as the framework to guide analysis in the study as it had been used effectively in previous qualitative studies to examine barriers and enable to post-ICU intervention implementation.^15^ Incorporating the domains of the TIDIER checklist enabled us to consider the benefits and limitations of different intervention design options.^16^ However, the data extracted from each article was limited to what was presented by study authors each publication, which may be biased by their analysis and interpretations.

## Conclusion

This review provides a framework for future research examining the optimal approach to support patient recovery after critical illness following hospital discharge. Implementation factors were identified relating to which components to include, who will deliver the intervention and how it will be delivered, and the impact of by patient, staff and setting factors. A key unanswered question is how any novel intervention interacts with and relies upon existing resources and care delivery, including the optimal approach to integrate with the patients’ primary care provider.

## Supplemental Material

sj-docx-1-inc-10.1177_17511437241308674 – Supplemental material for Factors to consider when designing post-hospital interventions to support critical illness recovery: Systematic review and qualitative evidence synthesis Supplemental material, sj-docx-1-inc-10.1177_17511437241308674 for Factors to consider when designing post-hospital interventions to support critical illness recovery: Systematic review and qualitative evidence synthesis by Jonathan Stewart, Ellen Pauley, Danielle Wilson, Judy Bradley, Nigel Hart and Danny McAuley in Journal of the Intensive Care Society
