# Supplementary material for: Factors to consider when designing post-hospital interventions to support critical illness recovery: Systematic review and qualitative evidence synthesis
Source: J Intensive Care Soc. 2025 Jan 3;26(1):80–95. doi: 10.1177/17511437241308674 (PMC11699563; doi:10.1177/17511437241308674)
Supplement: sj-docx-1-inc-10.1177_17511437241308674 – Supplemental material for Factors to consider when designing post-hospital interventions to support critical illness recovery: Systematic review and qualitative evidence synthesis [file sj-docx-1-inc-10.1177_17511437241308674.docx]

**Appendix 1: Summary of search terms**

| **Web of science** | **Embase** | **Ovid MEDLINE(R) ALL** | **EBSCO – CINAHL** |
| --- | --- | --- | --- |
| #1  intensive care*, or  ICU, or  critical care, or  crit* ill*  #2  enabler*, or  facilitate*, or  barrier*, or  implement*, or  qualitative, or  process evaluation  #3  post-discharge, or  home, or  discharge*, or  follow-up, or  follow up, or  post-ICU  #1 and #2 and #3 | S1 - intensive care unit/ or intensive care*.mp. or intensive care/  S2 -ICU.mp.  S3 - critical care.mp.  S4 - crit* ill*.mp.  S5 - 1 or 2 or 3 or 4  S6 - enabler*.mp.  S7 - facilitat*.mp.  S8 - barrier*.mp.  S9 - implement*.mp.  S10 - qualitative.mp. or qualitative analysis/ or qualitative research/  S11 - process evaluation.mp.  S12 - 6 or 7 or 8 or 9 or 10 or 11  S13 - post-discharge.mp.  S14 – home*.mp.  S15 - discharge*.mp.  S16 - follow-up.mp. or follow up/  S17 - post-ICU.mp.  S18 - 13 or 14 or 15 or 16 or 17  S19 - 5 and 12 and 18  S20 - limit 24 to (abstracts and conference abstracts)    S21 - 19 not 20 | S1 - intensive care unit/ or intensive care*.mp. or intensive care/  S2 -ICU.mp.  S3 - critical care.mp.  S4 - crit* ill*.mp.  S5 - 1 or 2 or 3 or 4  S6 - enabler*.mp.  S7 - facilitat*.mp.  S8 - barrier*.mp.  S9 - implement*.mp.  S10 - qualitative.mp. or qualitative analysis/ or qualitative research/  S11 - process evaluation.mp.  S12 - 6 or 7 or 8 or 9 or 10 or 11  S13 - post-discharge.mp.  S14 – home*.mp.  S15 - discharge*.mp.  S16 - follow-up.mp. or follow up/  S17 - post-ICU.mp.  S18 - 13 or 14 or 15 or 16 or 17  S19 - 5 and 12 and 18 | S1 - "process evaluation"  S2 - "qualitative"  S3 - "implement*"  S4 - "barrier*"  S5 - "facilitat*"  S6 - "enabler*"  S7 - "post-ICU"  S8 - "post-discharge"  S9 - "home*"  S10 - "discharge"  S11 - "follow-up"  S12 - "follow up"  S13 - "intensive care"  S14 - "critical care"  S15 - "crit* ill*"  S16 - "ICU"  S17 - S1 OR S2 OR S3 OR S4 OR S5 OR S6  S18 - S7 OR S8 OR S9 OR S10 OR S11 OR S12  S19 - S13 OR S14 OR S15 OR S16  S20 - S17 AND S18 AND S19 |

**Appendix 2: Summary of included studies**

| **First Author Year** | **Title** | **Country** | **Intervention** | **Study objective(s)** | **Study design** | **Participants** | **Sample size** | **Data collection**  **- methods** | **Data collection**  **- timing** | **Data analysis**  **- methods** |
| --- | --- | --- | --- | --- | --- | --- | --- | --- | --- | --- |
| Backman  2018 | Group meetings after critical illness: Giving and receiving strength | Sweden | Peer led support | Describe former intensive care patients’ feelings of sharing their experience of critical illness with other former patients | Primary qualitative | Adults, who had an ICU stay more than 96 hours, had visited the follow-up clinic at least one time, able to attend both group meetings, and ability to speak and write in Swedish. | 11 patients | Semi structured interviews  Notebook | Notebooks sent back within 14 days of group meeting.   Interviews 2-3 months after group meeting. | Inductive Content Analysis |
| Bench  2016 | Intensive care discharge summaries for general practice staff: a focus group study | UK | GP Information sharing / support | Understand the information needs of GP staff supporting patients from ICUs and their families after discharge from hospital and identifying the barriers/facilitators associated with providing ICU-primary care information. | Primary qualitative | Patients  Relatives  GP staff | 15 patients 4 relatives 20 GP staff | Focus groups  Semi structured interviews | June to September  2015 | Inductive thematic analysis  *(Newell and Burnard)* |
| Boehm, 2023 | Multidisciplinary ICU Recovery Clinic Visits: A Qualitative Analysis of Patient-Provider Dialogues. | USA | Telemedicine ICU follow-up clinic | Explore the content of dialogues between patients and clinicians in a multidisciplinary telemedicine ICU-RC. | Primary | Adults hospitalized in a medical or surgical ICU with septic shock or ARDS and projected to be discharged alive | 19 patients  13 caregivers | Observations (audio-recording of visits) | Telemedicine visits at 3 and 12 weeks after hospital discharge | Iterative inductive and deductive analysis |
| Castro-Avila 2021 | Support and follow-up needs of patients discharged from intensive care after severe COVID-19: a mixed-methods study of the views of UK general practitioners and intensive care staff during the pandemic's first wave. | UK | GP Information sharing / support  ICU follow-up services | Identify follow-up services that were available during and after the UKs first wave of the COVID-19 pandemic, early reflections on care during the first wave, and the views of critical care staff and general practitioners (GPs) about these patients’ future needs and care coordination. | Mixed methods | ICU staff (ICU consultants, senior nurses, rehabilitation coordinators) leading care for patients discharged from ICU  GPs | 17 ICU staff  6 GPs | Semi structured interviews | June to August 2020 | Thematic analysis |
| Clarke, 2023 | An Intensive Care Unit peer support group: Participants' views on format, content and the impact on recovery journeys. | UK | Peer Led Support | Understand how  participants experience the group, its format and outcomes using in-depth qualitative data. | Primary qualitative | ICU survivors with a 4 day or longer ICU admission | 11 patients | Semi structured  interview online or by telephone or were sent the interview schedule to provide a written response. | Following the Peer support group | Thematic Analysis *(Braun and Clarke)* |
| Clarke, 2024 | "It saved me": A thematic analysis of experiences of psychological therapy following critical illness and intensive care. | UK | Psychological Support | Explore patients experiences of psychological therapy as part of outpatient follow-up care. | Primary qualitative | Survivors with an ICU admission of 4 days or more at 3 months post-hospital discharge. | 20 patients | Semi structured interviews. | Following psychological therapy. | Thematic Analysis *(Braun and Clarke)* |
| **First Author Year** | **Title** | **Country** | **Intervention** | **Study objective(s)** | **Study design** | **Participants** | **Sample size** | **Data collection**  **- methods** | **Data collection**  **- timing** | **Data analysis**  **- methods** |
| Connolly  2014 | A UK survey of rehabilitation following critical illness: Implementation of NICE Clinical Guidance 83 (CG83) following hospital discharge | UK | Follow up and rehabilitation services | Characterise available follow-up and rehabilitation services, across the UK, and establish barriers to service provision. | Mixed methods | Senior respiratory critical care physiotherapy clinicians. | 182 physiotherapists | Open ended survey questions | Single dissemination of survey March 2013 with reminder to non-respondents six weeks later | ‘’Qualitative review of free-text comments made'' |
| Connolly  2021 | Recovery, rehabilitation and follow-up services following critical illness: an updated UK national cross-sectional survey and progress report | UK | Recovery, rehabilitation, and follow-up services | Comprehensively re-survey the current provision of recovery and follow-up services for adult critically ill patients across the UK to identify unmet areas of unmet need, inform service innovation and benchmark against clinical standards.'' | Mixed methods | Multi professional critical care clinicians delivering services at each site (Medics, nurses, physiotherapists)  Other (team lead, clinical director, ward manager)  Various (eg, clinical educator, audit lead)  Psychologist Dietitian | 176 professionals from various backgrounds | Open ended survey questions | Survey was open for completion for a period of 8 weeks.  Repeated circulation of the survey, including targeted approaches to non-responders where possible. | Summative content analysis |
| Engstrom  2008 | Re-visiting the ICU Experiences of follow-up visits to an ICU after discharge: A qualitative study | Sweden | Follow-up visit to ICU | Describe post-discharge follow-up visits to an ICU as experienced by both people who had been critically ill and cared for in an ICU and their close relatives. | Primary qualitative | Adults who had been critically ill, on respiratory treatment for at least 24 h, and cared for in an ICU for at least 72 h  Close relatives | 9 former patients  9 relatives | Narrative interviews | 3 and 9 months after their follow-up visit. | Thematic analysis |
| Engstrom  2010 | Critical care nurses experiences of follow-up visits to an ICU | Sweden | Follow-up visit to ICU | Describe critical care nurses experiences of follow-up visits by formerly critically ill people discharged from an ICU and their close relatives.'' | Primary qualitative | Critical Care Nurses who had regular experience of follow-up visits in an ICU in the northern part of Sweden. | 8 nurses | Interviews with a narrative approach. | During 2007 | Thematic content analysis  *(Downe-Wamboldt)* |
| Engstrom  2018 | Follow-up visit in an ICU: receiving a sense of coherence | Sweden | Follow-up visit to ICU | Describe patient’s experiences of a follow-up visit to an ICU after being critically ill and nursed there. | Primary qualitative | Adult patients admitted to the ICU for at least 72 h, attending a follow-up visit in the ICU. | 9 patients | Semi structured interviews | Interviews were carried out 2 months after ICU follow-up visit. | Content analysis |
| Ferguson  2019 | Patients Perceptions of an Exercise Program Delivered Following Discharge From Hospital After Critical Illness (the Revive Trial) | UK | Follow-up programme:  Physical Exercise / Physiotherapy | Explore patients satisfaction with the REVIVE trial exercise  program. | Primary qualitative | Adult patients from the exercise intervention group of the REVIVE trial from 6 ICUs in Northern Ireland.  • Aged ≥18 years  • Mechanical ventilation >96 hours  • Planned to be discharged home  • Medically fit to participate  • Not participating in another rehabilitation programme | 21 patients  (of 30 allocated to intervention arm) | Semi structured interviews | 1 individual face-to-face interview, or via telephone if necessary, at the 6-month follow-up time point. | Thematic content analysis  *(Burnard)* |
| **First Author Year** | **Title** | **Country** | **Intervention** | **Study objective(s)** | **Study design** | **Participants** | **Sample size** | **Data collection**  **- methods** | **Data collection**  **- timing** | **Data analysis**  **- methods** |
| Gehrke-Beck 2021 | General practitioners' views and experiences in caring for patients after sepsis: a qualitative interview study. | Germany | Patient monitoring  Patient education  Educational outreach for GPs | Describe GPs views and experiences of caring for postsepsis patients and of participating a specific outreach training, in order to inform and contribute to applicable future aftercare structures in primary care. | Primary qualitative | GPs who were participants at the Berlin trial site of the SMOOTH trial | 14 GPs | Semi structured interviews | January to August 2013 | Thematic analysis |
| Glaeemose, 2024 | Peer support in intensive care unit follow-up: A qualitative evaluation. | Denmark | Peer Led Support | Evaluate the content and setting of the café meetings  and to explore participants' experiences of meeting peers. | Primary qualitative | Adult patients hospitalized in one of the four ICUs who had participated in an individual follow-up consultation and in a café meeting.  Relatives participating in a café meeting. | Observations:  77 participants  (42 ICU survivors and 35 relatives)  Interviews:  22 participants  (12 patients and 10 relatives) | Focused ethnographic observations.  Semi-structured interviews | Ethnographic observations during café meetings.  Interviews 5–21 days after café meetings | Thematic analysis *(Braun & Clarke)* |
| Griffiths  2006 | A national survey of intensive care follow-up clinics | UK | Follow-up clinics | Provide an estimate of the number of ICU follow-up clinics and details of how they are structured, run and funded. | Mixed methods | Lead clinicians or head nurses in ICUs providing level 2 or 3 care to patients in the UK | 266 lead clinicians or nurses | Open ended survey questions | Single time point | Unclear |
| Haines  2019 | Enablers and Barriers to Implementing ICU Follow-Up Clinics and Peer Support Groups Following Critical Illness: The Thrive Collaboratives. | International collaborative: United States of America, United Kingdom, and Australia.  Sites involved in Society of Critical Care Medicine (SCCM) THRIVE program | Follow-up clinics  Peer led support | Discover enablers that helped hospital-based clinicians establish post-ICU clinics and peer support programs, and identify barriers that challenged them.'' | Primary qualitative | Members of the Society of Critical Care Medicine (SCCM) Thrive collaboratives  Clinicians from 21 sites- participants attending the in-person Congress meetings, representing various international sites (the United States, the United Kingdom, Australia) and professions (nursing, medical, allied health). | ICU follow-up clinic collaborative n = 15 Medical 8  Nursing 2  Pharmacist 4  Allied health 1  Peer support collaborative n = 11 Medical 6  Nursing 4 Pharmacist 1 Allied health 2 | Focus groups  Semi structured interviews | Conducted at international Society of Critical Care Medicine (SCCM) congress | Thematic analysis *(Braun & Clarke, 2006)* |
| Hanifa  2018 | Picking up the pieces: Qualitative evaluation of follow-up consultations post intensive care admission. | Denmark | Follow-up consultation | Describe former ICU patient’s consultation experiences, specifically regarding content and setting.  Explore the benefits of the consultation in regard to individual patients symptoms of PICS. | Primary qualitative | Patients in the ICU for at least five days and mechanically ventilated for at least 24 h during hospitalisation | 10 patients | Semi structured interviews  Observations | Field notes during follow-up consultation  Interview 2-4 weeks after consultation | Focus Ethnography   Hermeneutic-phenomenological approach |
| **First Author Year** | **Title** | **Country** | **Intervention** | **Study objective(s)** | **Study design** | **Participants** | **Sample size** | **Data collection**  **- methods** | **Data collection**  **- timing** | **Data analysis**  **- methods** |
| Hauschildt  2022 | Hospital Discharge Summaries Are Insufficient Following ICU Stays: A Qualitative Study. | United States of America | GP Information sharing / support | To understand how Primary care professionals perceive the discharge summaries they routinely receive for patients who were critically ill, and what additional information they would like to know to better support their patients recovery following critical illness. | Primary qualitative | Primary care physicians associated with an academic health system who treat patients in the systems primary care clinics and at the associated Veteran Affairs Medical Center. | 14 primary care physicians | Semi structured interviews | Between September 2020 and April 2021 | Modified version of the Rigorous and Accelerated Data Reduction  thematic memoing |
| Henderson 2022 | Evaluation of a health and social care programme to improve outcomes following critical illness: a multicentre study. | United Kingdom (Scotland) | ICU follow-up programme | An embedded process evaluation to assess factors influencing implementation of the InS:PIRE programme. | Mixed methods | Multidisciplinary team members from all five sites who implemented the InS:PIRE programme | Unclear | Via in-person learning sessions | Twice a year across the implementation and evaluation period (2.5 years). | Unclear |
| Jensen  2019 | Intervention fidelity in postintensive care follow-up consultations at ten sites in the RAPIT-trial: A mixed-methods evaluation. | Denmark | Follow-up consultation  Physical rehabilitation offered | Evaluate the intervention fidelity of study design, provider training, delivery, receipt, and enactment. | Mixed methods | Post-ICU patients and ICU nurses from 10 Danish ICUs who participated in the RAPIT trial | Nurse focus groups  - 3 patients  Consultations  - 12 patients  Interviews  - 14 nurses | Focus groups  Transcribed  interviews data  (audio‐recordings of consultations)  Exit semi‐structured  interviews | During and after the RAPIT-trial in 2012 - 2017. | Deductive-inductive thematic analysis *(Braun & Clarke, 2006)* |
| Kovaleva, 2023 | Patient and caregiver experiences with a telemedicine intensive care unit recovery clinic. | United States of America | ICU follow-up clinic | Explore patient and care  giver experiences with a telemedicine ICU recovery clinc. | Primary qualitative. | Patient:   - >=18 years - English-speaking - Without visual, hearing, or cognitive impairments precluding participation - ICU stay with sepsis and/or acute respiratory distress syndrome diagnosis; - electronic device with internet connection; - basic computer skills - completed at least one telemedicine ICU-RC visit.   Caregivers   - >=18 years and English-speaking; - providing unpaid patient care; - willing to comment on the telemedicine ICU-RC visit(s) | 14 patients  12 caregivers | Semi-structures interviews via telephone. | After the 12-week visit. | Conventional content analysis |
| **First Author Year** | **Title** | **Country** | **Intervention** | **Study objective(s)** | **Study design** | **Participants** | **Sample size** | **Data collection**  **- methods** | **Data collection**  **- timing** | **Data analysis**  **- methods** |
| Leggett, 2023 | Fragmentation of care between intensive and primary care settings and opportunities for improvement | Australia | GP Information sharing / support | Explore the current gaps in care across the transitions from the ICU to primary care—to identify opportunities for health system improvements | Primary qualitative | Patients and carers:   - Adults>18 years, mechanically ventilated for >24 hours. - Access to computer/tablet with microphone, camera & internet, or smartphone - English speaking - Without cognitive or neurological limitation that would preclude interview participation.   Intensivist and GPs:   - Recruited within working networks at participating hospital sites | 46 interviews  - 15 patients  - 8 caregivers  - 15 intensivists  - 8 GPs | Semi-structured interviews | Following hospital discharge. | Framework analysis |
| Leggett, 2024 | Clinician and patient identified solutions to reduce the fragmentation of post-ICU care in Australia. | Australia | GP Information sharing / support | Identify potential  solutions to improve the communication between  treating teams and integration of care following an  ICU admission. | Primary qualitative | As above. | 46 interviews  - 15 patients  - 8 caregivers  - 15 intensivists  - 8 GPs | Semi-structured interviews | Following hospital discharge. | Framework analysis |
| Major  2021 | Feasibility of a home-based interdisciplinary rehabilitation program for patients with Post-Intensive Care Syndrome: the REACH study. | Netherlands | Follow-up programme   - Physical Exercise - Dietician - Occupational Therapy | Investigate the feasibility of an interdisciplinary home-based intervention for patients with new or worsened impairments within one of the domains of PICS, initiated immediately after hospital discharge and targeting (physical) recovery and self-management in comparison to patients receiving usual care.'' | Mixed methods | REACH professionals | 11 professionals | Focus groups  Open ended survey questions | End of the REACH study | Thematic analysis |
| McPeake  2017 | Intensive Care Syndrome: Promoting Independence and Return to Employment (InS:PIRE). Early evaluation of a complex intervention | UK | Follow-up programme   - Physiotherapy - Psychological support - Medication review - Social support - Peer led support | Understand the impact of a complex intervention for ICU survivors | Mixed methods | Patients and caregivers who took part in the Intensive Care Syndrome: Promoting Independence and Return to Employment (InS:PIRE) programme | 11 | Semi structured interviews | 6 months | Content analysis  *(Burnard)* |
| **First Author Year** | **Title** | **Country** | **Intervention** | **Study objective(s)** | **Study design** | **Participants** | **Sample size** | **Data collection**  **- methods** | **Data collection**  **- timing** | **Data analysis**  **- methods** |
| McPeake  2020 | Key Components of ICU Recovery Programs: What Did Patients Report Provided Benefit? | International collaborative: United States, United Kingdom, and Australia.  Sites involved in Society of Critical Care Medicine (SCCM) THRIVE programme. | ICU recovery programmes | Understand what the most highly beneficial components of an ICU recovery program were from a patient perspective. | Primary qualitative | Adult patients older who had a critical care experience | 66 patients  - 52 had used an ICU recovery program  - 14 had not | Semi structured interviews | Length of time since ICU discharge, n (%) < 6 mo = 15 (22.8) 7-11 mo = 9 (13.6) 1-2 yr = 12 (18.2) 2-5 yr = 22 (33.3) > 5 yr = 8 (12.1) | Thematic content analysis  *(Miles and Huberman)* |
| Parker  2020 | Usability and acceptability of a mobile application prototype for a combined behavioural activation and physical rehabilitation intervention in acute respiratory failure survivors. | United States | Mobile app:   - Physical Exercise / Physiotherapy - Psychological support | Assess user experience, including usability and acceptability, of an App prototype to deliver a combined behavioural activation and rehabilitation intervention to survivors of acute respiratory failure. | Mixed methods | Acute respiratory failure survivors and care partners  Adults acute respiratory failure survivors, who required mechanical ventilation >24 h , at least mild depression symptoms before hospital discharge, access to an internet-enabled technology device at home, and ability to use a technology device. | 10 patients | Semi structured interviews | Contacted by phone soon after arriving home to schedule an in-person home visit with both the survivor and care partner present. | Themes were established a priori, while the remaining themes were identified through thematic analysis |
| Pattison  2007 | After critical care: a study to explore patients' experiences of a follow-up service. | UK | Follow-up service | Explore experiences of follow-up inpatient visits and a nurse-led outpatient clinic for patients who have undergone major cancer surgery requiring critical care. | Mixed methods | Patients over the age of 18 years, who had undergone major cancer surgery (for sarcoma or upper gastro-intestinal) and had spent at least 48 hours in the critical care unit. | 27 patients | Unstructured interviews  Open ended survey questions | Three and six months following discharge | Grounded theory |
| Petersson  2011 | Patients participation in and evaluation of a follow-up program following intensive care | Sweden | Follow-up programme | Explore and describe patient’s participation in and evaluation of a follow-up program at a nurse-led clinic (NLC). | Mixed methods | Patients attending a Nurse Led Clinic | Patients  Contact n=68 2-month n=57 6-month n=49 | Open ended survey questions | A few days after discharge from the ICU (visit on the ward), 2 months and 6 months. | Open ended questions were grouped in relation to their content  *(Knafl & Webster)* |
| Prevedello 2021 | The burden of implementation: A mixed methods study on barriers to an ICU follow-up program. | Belgium | Follow-up programme | Evaluate and explore the barriers to implementation from the perspective of the healthcare provider. | Mixed methods | Staff members involved in the ICU program | 12  - 6 nurses  - 1 psychologist  - 2 physiotherapists  - 2 doctors  - 1 social worker | Semi structured interviews  Observations | Between May & December 2018. | Thematic content analysis guided by the principles of grounded theory |
| **First Author Year** | **Title** | **Country** | **Intervention** | **Study objective(s)** | **Study design** | **Participants** | **Sample size** | **Data collection**  **- methods** | **Data collection**  **- timing** | **Data analysis**  **- methods** |
| Prinjha  2009 | What patients think about ICU follow-up services: a qualitative study | UK | Follow-up services | Explores patients' perceptions and experiences of these services. | Primary qualitative | Patients: Men and women from across the UK of different age groups and social and ethnic backgrounds. It also included participants who were admitted to the ICU as emergency and elective admissions, for different lengths of stay. | 40 patients | In-depth narrative interviews | During 2005 | Grounded theory |
| Rohr  2021 | Experiences, opinions and expectations of health care providers towards an intensive care unit follow-up clinic: Qualitative study and online survey. | Germany | Follow-up clinic | Explore the experiences, opinions and expectations of health care providers regarding an ICU follow-up clinic in Germany. | Mixed methods | Healthcare professionals from intensive care, nursing, neurology, psychology, physiotherapy, speech therapy and general practice from both the inpatient and outpatient settings | 47 professionals  Doctor 16  Nurse 11  Physiotherapist 8  Psychologist 3  Speech therapist 3  Social worker 2 Administrative 2 Occupational therapist 1 Pastoral worker 1 | Focus groups  Semi structured interviews | Unclear | Thematic content analysis |
| Sanftenberg, 2023 | Barriers and opportunities for implementation of a brief psychological intervention for post-ICU mental distress in the primary care setting - results from a qualitative sub-study of the PICTURE trial. | Germany | Psychological Therapy  - Narrative exposure therapy (NET) | Analyse the role of the GP team concerning the practical implementation of the narrative exposure therapy (NET) assessed by participating patients within the PICTURE  trial. | Primary qualitative | Ten patients from the intervention group of the  PICTURE trial  Trial participants were adult patients with a total score of at least 20 points on the post traumatic diagnostic scale (PDS-5) | 10 patients | Semi-structured interview via telephone | Following the three scheduled NET sessions. | Qualitative content analysis  *(Mayring)* |
| vanBeusekom 2018 | Lessons learnt during the implementation of a web-based triage tool for Dutch intensive care follow-up clinics. | Netherlands | Web-based triage tool for screening symptoms of post-intensive care syndrome for a ICU follow-up clinic | Evaluate the feasibility of our web-based triage tool in the ICU follow-up clinic and to assess the outcomes gained by web-based questionnaires compared with those from conventional paper based questionnaires. | Mixed methods | Healthcare professionals who worked with the system | 5 intensivists  6 ICU nurses  1 physical therapist  2 secretaries | Semi structured interviews | During 2015  Interviews conducted after finishing the pilot study. | Thematic content analysis |
| **First Author Year** | **Title** | **Country** | **Intervention** | **Study objective(s)** | **Study design** | **Participants** | **Sample size** | **Data collection**  **- methods** | **Data collection**  **- timing** | **Data analysis**  **- methods** |
| Walker  2015 | Project Post Intensive Care eXercise (PIX): A qualitative exploration of intensive care unit survivors perceptions of quality of life post-discharge and experience of exercise rehabilitation | UK | Physical Exercise / Physiotherapy | Gain a more in-depth insight into patients perceptions of their quality of life after hospital discharge and their experiences of aftercare services, be it usual care or the PIX exercise intervention programme. | Primary qualitative | Participants in the Project Post Intensive Care eXercise (PIX) study  Adult patients admitted to the ICU of one of two large teaching hospitals, had received a minimum of 3 days of ventilator support (for the emergency management of trauma or sepsis), had been discharged home within 6 months of hospital admission. | 16 patients  (8 underwent the supervised exercise programme) | Focus groups | Following intervention delivery | Thematic analysis  *(Braun and Clarke)* |
| Zhang, 2023 | Barriers and facilitators to offering post-intensive care follow-up services from the perspective of critical care professionals: A qualitative study. | China | ICU follow-up services | Explore and describe the barriers and facilitators of post-intensive care follow-up services from the perspective of critical  care professionals. | Primary qualitative | ICU medical staff engaged in follow-up services   - Consented to participate - Had worked in the ICU for at least 3 years - Formal employee of a hospital. | 21 ICU medical staff | Semi-structured interviews | August - December 2022 | Qualitative content analysis  *(Graneheim, 2004)* |
| Zilahi  2019 | Information sharing between intensive care and primary care after an episode of critical illness; A mixed methods analysis. | Ireland | Information sharing with GP | Evaluate information sharing in Ireland between ICU medical staff and GPs around an episode of critical illness.  Explore the factors affecting both the quality of information sharing as well as how the information is utilised by GPs when received. | Mixed methods | Intensive care medicine consultants (ICMCs) in the Intensive Care Society of Ireland database (comprising >90% of ICMCs in Ireland)  GPs from the Irish College of General Practitioners (ICGP) database (includes 85% of GPs in Ireland) | Survey (n=163):  - 65 ICMCs  - 97 GPs  Interview (n=13):  - 5 ICMCs  - 8 GPs | Semi structured interviews  Open ended survey questions | May - July 2016 | Directed content analysis  *(Hsieh)* |

**Appendix 3 – Summary of included interventions (TIDieR checklist domains)**

| **First Author**  **Year** | **WHAT - Type** | **What - content** | **WHO - Who delivered the intervention** | **WHO – Who received the intervention** | **HOW - Delivery** | **WHERE - Setting (Location where intervention delivered)** | **WHEN - Start time point** | **HOW MUCH**  **Duration & Intensity** |
| --- | --- | --- | --- | --- | --- | --- | --- | --- |
| Backman 2018 | Peer led support | Discussion of ICU stay, ward stay, and recovery at home. | Nurse - ICU | Patients treated in a general ICU at a regional Hospital, in the previous 3–18 months and met the inclusion criteria:   - Adults over 18 years old - ICU stay more than 96 hours - Visited a follow-up clinic at least one time - Able to attend both group meetings - Ability to speak and write in Swedish | In-person | Hospital outpatient | All patients who had been treated in a general ICU at a regional Hospital, in the previous 3–18 months | Two group meetings, one month apart. |
| Bench 2016 | ICU discharge summaries for general practice staff | Variable between focus group & interview participants | ICU staff | GPs and practice nurses from one inner London NHS Clinical Commissioning Group | NA – ICU to primary care information transfer | NA – ICU to primary care information transfer | At hospital discharge | NA – ICU to primary care information transfer |
| Boehm, 2023 | Telemedicine ICU recovery visits | Identified and addressed long term cognitive, physical, mental health, and socioeconomic | ICU pharmacist  ICU physician  Psychologist | Inclusion: Adults hospitalized in a medical or surgical ICU with septic shock or ARDS and projected to be discharged alive  Exclusion: No computer, electronic device, or connectivity for telemedicine. Out-of state residence; hospice referral; acute neurologic injury; or preexisting severe substance abuse, psychiatric disorder, or dementia. | Virtual | Telemedicine | Initial visit at 3 weeks. | Visits at 3 and 12 weeks after hospital discharge |
| Castro-Avila 2021 | ICU-primary care information sharing  ICU follow-up services | Variable between survey & interview participants | Variable between survey & interview participants | Variable between survey & interview participants | Variable between survey & interview participants | Variable between survey & interview participants | Variable between participants  All unit staff we interviewed follow patients up 2-3 months after ICU discharge, but a minority also routinely call patients weekly. | Variable between participants  All unit staff we interviewed follow patients up 2-3 months after ICU discharge, but a minority also routinely call patients weekly |
| Clarke, 2023 | Peer led support | Share experiences, a means to better understand experiences, and opportunity for reciprocity in giving and receiving support | Facilitated y critical care psychologist, supported by a critical care rehabilitation nurse. | Psychological screening questionnaires are sent 3 months post-discharge to ICU survivors with a 4 day or longer ICU admission. Group recruitment is from those attending the follow-up clinic or assessment appointment. | Prior to Covid-19, the group ran monthly in-person for 2 hours but transitioned to online fortnightly for 1 hour. | Virtual | >3months post-discharge | Fortnightly |
| **First Author**  **Year** | **WHAT - Type** | **What - content** | **WHO - Who delivered the intervention** | **WHO – Who received the intervention** | **HOW - Delivery** | **WHERE - Setting (Location where intervention delivered)** | **WHEN - Start time point** | **HOW MUCH**  **Duration & Intensity** |
| Clarke, 2024 | Psychological Therapy | Evidenced based therapy including Cognitive Behavioral Therapy (CBT), Acceptance and Commitment Therapy  (ACT), and Eye Movement Desensitization and Reprocessing (EMDR). | Critical Care Psychologist | Survivors with an ICU admission of 4 days or more at 3 months post-hospital discharge with screening questionnaire scores indicating psychological distress. | In-person and remote | Unknown | >3 months post hospital discharge. | The range of psychotherapy sessions undertaken  by participants was 1–29; the average was 12. |
| Connolly 2014 | Rehabilitation following critical illness  Variable between survey respondents  *(Summary in article)* | Variable between survey respondents  *(Summary in article)* | Variable between survey respondents  *(Summary in article)* | Variable between survey respondents | Variable between survey respondents  *(Additional information in supplementary materials)* | Variable between survey respondents  *(Additional information in supplementary materials)* | Variable between survey respondents  *(Additional information in supplementary materials)* | Variable between survey respondents  *(Additional information in supplementary materials)* |
| Connolly 2021 | Recovery, rehabilitation and follow-up services following critical illness  Variable between survey respondents | Variable between survey respondents  *(Summary in article)* | Variable between survey respondents  *(Summary in supplementary materials)* | Variable between survey respondents | Variable between survey respondents | Variable between survey respondents  *(Summary in supplementary materials)* | Variable between survey respondents | Variable between survey respondents  *(Summary in article)* |
| Engstrom 2008 | Follow-up visit to the ICU | Review of ICU stay  ICU room visit  Patient diary review | Doctor – ICU  Nurse – ICU | Person had been critically  ill, on respiratory treatment for at least 24 hours, and cared for in an ICU for at least 72 hours | In-person | ICU ward | About 6 months after leaving the ICU | Single visit |
| Engstrom 2010 | Follow-up visit to the ICU | Review of ICU stay  ICU room visit  Patient diary review | Doctor – ICU  Nurse – ICU | Person had been critically  ill, on respiratory treatment for at least 24 hours, and cared for in an ICU for at least 72 hours | In-person | ICU ward | About 6 months after leaving ICU | Single visit |
| Engstrom 2018 | Follow-up visit to the ICU | Review of ICU stay  ICU room visit  Patient diary review | Doctor – ICU  Nurse - ICU | Person had been critically ill and cared for in an ICU for at least 72 hours | In-person | ICU ward | About 3 months after leaving the hospital | Single visit |
| Ferguson 2019 | Follow-up programme | Physical Exercise / Physiotherapy | Physiotherapist – ICU  *(From original paper - McDowell, 2016)* | Participants in the REVIVE trial from general intensive care units  (ICUs) in six hospitals in Northern Ireland, UK;   - Aged ≥18 years - Mechanical ventilation >96 hours - Planned to be discharged home - Medically fit to participate - Not participating in another rehabilitation programme (cardiac or pulmonary rehabilitation) | Hybrid (remote and in-person) | Supervised sessions  Hospital gymnasium or Patient home  Unsupervised sessions  Patents home  *(From original paper - McDowell, 2016)* | Ideally within 2 weeks of hospital discharge or when the patient was deemed medically fit and/or able to attend)  *(From original trial paper - McDowell, 2016)* | Two supervised and one unsupervised exercise session/s per week for 6 weeks  *(From original trial paper - McDowell, 2016)* |
| **First Author**  **Year** | **WHAT - Type** | **What - content** | **WHO - Who delivered the intervention** | **WHO – Who received the intervention** | **HOW - Delivery** | **WHERE - Setting (Location where intervention delivered)** | **WHEN - Start time point** | **HOW MUCH**  **Duration & Intensity** |
| Gehrke-Beck 2021 | Follow-up consultation  GP education | Structured aftercare  programme in general practice for sepsis survivors   - Discharge management - Training of GPs and patients in sepsis sequelae and evidence-based treatment options - Patient monitoring   (From original paper, Schmidt, 2014) | Specialised nurse ‘Case manager’  Liaison physician – a GP trained in sepsis aftercare | General Practitioners  Patient participants (sepsis survivors) at the Berlin trial site of the SMOOTH trial | Hybrid (remote and in-person) | Educational visit in the GP practice  Hospital  Patient home  General practice | After discharge from the ICU, intervention patients receive specific discharge forms from the case manager.  *(From original trial paper, Schmidt, 2014)* | One outreach educational visit after the patient’s discharge according to time preferences of the GPs.  Patients contacted every month during the first six months after discharge from ICU and every three months during months 7 to 12 for the monitoring.  *(From original trial paper, Schmidt, 2014)* |
| Glaeemose, 2024 | Peer Led Support | Peer led support | Nurse | Adult patients hospitalized in one of the four ICUs who sign up.  Relatives participating in a café meeting. | In-person | In the hospital. | Held twice a year. | Unclear. |
| Griffiths 2006 | Follow-up clinics | Variable between respondents  *(Summary in paper)* | Variable between respondents | Variable between respondents | Variable between respondents | Variable between respondents | Variable between respondents | Variable between respondents |
| Haines 2019 | Follow-up clinics  Peer led support | Variable between sites | Variable between sites | Variable between sites | Variable between sites | Variable between sites | Variable between sites | Variable between sites |
| Hanifa 2018 | Follow-up consultation | Review of ICU stay  Reunion with ICU staff  Onward referral | Nurse | Patients in the ICU for at least five days and mechanically ventilated for at least 24 h during hospitalisation | In-person | ICU where patient was admitted | 3 months post ICU admission | Single session, 1 hour |
| Hauschildt, 2022 | GP Information sharing / support | Hospital discharge summaries for patients who had been critically ill | Staff at large, academic medical center | Primary Care Physicians | Unclear | Large, academic medical center | Participants were asked  to recall hospital discharge summaries from hospitals  within their system | NA - Hospital discharge summary |
| Henderson, 2022 | Follow-up programme | Follow-up programme;   - Physiotherapy - Psychological support - Medication review - Social / economic support | Doctor - ICU  Nurse - ICU  Physiotherapist  Pharmacist  Neuropsychology  Social worker | - Level 3 care (multiple organ support and/or invasive respiratory support) or - More than 7 days of level 2 care (single organ support or postoperative care) | In-person | Hospital outpatient | 4- 12 weeks after  hospital discharge | Weekly for 5 weeks, with return appointments at 3  months and 12 months. |
| **First Author**  **Year** | **WHAT - Type** | **What - content** | **WHO - Who delivered the intervention** | **WHO – Who received the intervention** | **HOW - Delivery** | **WHERE - Setting (Location where intervention delivered)** | **WHEN - Start time point** | **HOW MUCH**  **Duration & Intensity** |
| Jensen 2019 | Follow-up consultations | Photographs from ICU  Reflection sheets  Information pamphlet  Communication | Nurse | Ten Danish ICUs participated in the RAPIT‐trial. Participants eligible if   - Danish-speaking - Adults (≥18 years) - Mechanically ventilated ≥48 hours - Did not meet criteria for baseline dementia. | Hybrid (remote and in-person) | Hospital outpatient | First consultation 1-3 months post-ICU. | 3 consultations: 1-3 months, 5 months and 10 months post ICU  (From Supplementary materials) |
| Kovaleva, 2023 |  | Medication reconciliation  Medical exam  ICU debrief  Cognitive screening | Pharmacist  ICU physician  Neuropsychologist | Patient:   - >=18 years - English-speaking - Without visual, hearing, or cognitive impairments precluding participation - ICU stay with sepsis and/or acute respiratory distress syndrome diagnosis; - electronic device with internet connection; - basic computer skills - completed at least one telemedicine ICU-RC visit. | Remote videoconferences | From a hospital outpatient exam room | 3 weeks post hospital discharge. | Two sessions 3- and 12-weeks post hospital discharge |
| Leggett, 2023 | Fragmentation of care between intensive and primary care settings and opportunities for improvement | Hospital discharge communication | Intensivist and GPs:   - Recruited within working networks at participating hospital sites | Patients and carers:   - Adults>18 years, mechanically ventilated for >24 hours. - Access to computer/tablet with microphone, camera & internet, or smartphone - English speaking - Without cognitive or neurological limitation that would preclude interview participation.   Intensivist and GPs:   - Recruited within working networks at participating hospital sites | Variable between participants | Variable between participants | Variable between participants | Variable between participants |
| Leggett, 2024 | Clinician and patient identified solutions to reduce the fragmentation of post-ICU care in Australia. | Hospital discharge communication | As above | As above | As above | As above | As above | As above |
| **First Author**  **Year** | **WHAT - Type** | **What - content** | **WHO - Who delivered the intervention** | **WHO – Who received the intervention** | **HOW - Delivery** | **WHERE - Setting (Location where intervention delivered)** | **WHEN - Start time point** | **HOW MUCH**  **Duration & Intensity** |
| Major 2021 | Follow-up programme | Physical Exercise  Dietician  Occupational Therapy | Nurse  Physiotherapist  Dietician Occupational Therapist | Recruited from 2 university and 5  general hospitals in the Amsterdam area, the Netherlands.  Eligible if;   - Received mechanical ventilation (MV) of ≥ 48 h in the ICU - Had developed new or worsened impairments during or after the ICU-stay unrelated to the initial admission diagnosis - discharged home with an indication for physical therapy (PT) - No serious (preexisting) cognitive and/or psychiatric impairments hindering compliance to the physical tests - Adequate understanding of the Dutch or English language. | In-person | Physiotherapy: Patient home, then nearby physiotherapy practice when physical condition allowed | 1 week after hospital discharge | ‘’Frequency of sessions averaged 2 half hour sessions per week in phase 1 and 30- to 60-min sessions twice a week in phase 2. In phase 3 participants often trained independently with irregularly scheduled supervised exercise sessions, as deemed necessary. The total duration of the REACH intervention was not specified a priori as decision-making depended on individual patient needs.’’ |
| McPeake 2017 | Follow-up programme | Peer led support  Physical Exercise  Psychological support  Medical review  Social / economic support | Doctor – ICU  Nurse - ICU  Physiotherapist  Pharmacist  Clinical psychologist | 20-bed mixed medical/surgical critical care unit in Glasgow Royal  Infirmary (GRI).  Eligible if;   - Adults of Working age (>18 years and <65 years) - Level-three stay of greater than 72 hours, or a level-two stay of greater than two weeks - No significant brain injuries | In-person | Hospital outpatient | Invited to attend between 6-20 weeks post hospital discharge | Five week programme  ‘’Weekly physiotherapy class’’  ‘’During the first three weeks, each patient and caregiver also received an individual appointment with nursing and medical staff, the pharmacist and the physiotherapist.’’  ‘’Over the fourth and fifth weeks patients and caregivers had group sessions with their peers.’’  ‘’final (fifth) week; the social prescription week’’ |
| McPeake 2020 | ICU recovery programmes | Physical therapy  Cognitive support  Peer support  Caregiver interventions  Socio-economic support  Psychological support  Information on short- and long-term adaption | Variable between participants | - Patients older than 18 years - Critical care experience - Adequate English language - No ongoing severe neurologic/ cognitive impairment - No continued inpatient status in a hospital or rehabilitation setting.   . | Variable between respondents  Online (virtual) = 15.2% In person = 63.6% No support group = 14% | Variable between participants | Variable between participants | Variable between participants |
| **First Author**  **Year** | **WHAT - Type** | **What - content** | **WHO - Who delivered the intervention** | **WHO – Who received the intervention** | **HOW - Delivery** | **WHERE - Setting (Location where intervention delivered)** | **WHEN - Start time point** | **HOW MUCH**  **Duration & Intensity** |
| Parker 2020 | Behavioural activation and physical rehabilitation  eHealth | Physical Exercise  Psychological support | Research staff members | - Adult acute respiratory failure survivors - Required mechanical ventilation >24 h - At least mild depression symptoms before hospital discharge - Access to an internet-enabled technology device at home, and ability to use a technology device. | Hybrid (remote and in-person)  Mobile app | Patient home | Contacted, by phone, soon after arriving home to schedule an in-person home visit with both the survivor and care partner present. | Unclear |
| Pattison 2007 | Follow-up clinic | Unclear | Nurse | - Age over 18 years, - Undergone major cancer surgery (for sarcoma or upper gastro-intestinal) - Spent at least 48 hours in the critical care unit | In-person | Hospital outpatient | ''timed to correspond with their first surgical review as an outpatient'' | ''a nurse-led appointment, timed to correspond with their first surgical review as an outpatient, and again at six months.'' |
| Petersson 2011 | Follow-up clinic | Information provision  ICU visit  Referral to social worker, psychologist of clergyman | Nurse | - Adult over 18 years - ICU stay over 72 h | Hybrid (remote and in-person) | Hospital outpatient  (Nurse led clinic located close to ICU) | During hospital admission | Duration: 3 - 6 months  3 visits: A few days after ICU discharge, 2 months and 6 months |
| Prevedello 2021 | Follow-up clinic | Physiotherapy  Medical/nurse review  Psychology assessment | Doctor – ICU  Nurse - ICU  Physiotherapist  Psychologist  Social worker | - Adults ≥18 years - At least five days in the ICU - No language barrier - No transfer to another institution - No neurological sequelae, social problems, and pregnancy. | In-person | Unclear | During ICU admission | Duration: 3 - 6 months  Four time points: ICU discharge, hospital discharge, and 3 and 6 months after ICU discharge. |
| Prinjha 2009 | Follow-up services | Variable depending on the patient | Variable depending on the patient | Former ICU patients from the UK | Variable depending on the patient | Variable depending on the patient | Variable depending on the patient | Variable depending on the patient |
| Rohr 2021 | Follow-up clinic | Development phase | Development phase | Development phase | Development phase | Development phase | Development phase | Development phase |
| Sanftenberg, 2023 | Psychological therapy | brief NET-oriented primary care  intervention combined with systematic trauma monitoring in ICU survivors | General Practitioner  Medical assisstant | - Adults aged 18 to 85 years - Mechanical ventilation ≥3 days - Sequential Organ Failure Assessment (SOFA) score ≥ 5 - Life expectancy ≥9 months (as assessed by the intensive care physician) - Primary Care PTSD Screen score ≥3   *(From trial protocol, Gensichen, 2018)* | Hybrid (remote and in-person) | Primary care physician practice | Baseline assessment 3 months after ICU discharge  NET sessions start 4 weeks thereafter  *(From trial protocol, Gensichen, 2018)* | Three sessions each 30 minutes |
| **First Author**  **Year** | **WHAT - Type** | **What - content** | **WHO - Who delivered the intervention** | **WHO – Who received the intervention** | **HOW - Delivery** | **WHERE - Setting (Location where intervention delivered)** | **WHEN - Start time point** | **HOW MUCH**  **Duration & Intensity** |
| vanBeusekom 2018 | Follow-up clinic | Web-based triage tool for symptoms of postintensive care syndrome  Review of questionnaires and referral to a specialist if necessary | All ICUs participating in the Dutch National  Intensive Care Evaluation (NICE) registry that had an ICU follow-up clinic | ICU survivors who had been admitted to an ICU participating in the Dutch National Intensive Care Evaluation (NICE) registry that had an  ICU follow-up clinic | In-person | ICU follow-up clinics aligned to ICUs participating in the Dutch National  Intensive Care Evaluation (NICE) registry | ''9 weeks after hospital discharge, the health professionals received a prompt to send the survivor an invitation by email to fill out a set of online questionnaires and to invite the survivor to visit the ICU follow-up clinic 3 months after hospital discharge.'' | Single follow-up appointment after questionnaire |
| Walker 2015 | Follow-up programme | Physical Exercise | Physiotherapist  From Original paper (Batterham, 2014) | Participants in the PIX study  (Batterham, 2014)  Patients admitted to the ICU of one of two large teaching hospitals were invited to participate.  Eligible patients were;   - Aged 18–65 yr - Received a minimum of 3 days of ventilator support (for the emergency management of trauma or sepsis) - Discharged home within 6 months of hospital admission. - Able to climb a flight of stairs, - Not enrolled in another rehabilitation programme - No medical contraindication to cardiopulmonary exercise testing | Hybrid (remote and in-person)  From Original paper (Batterham, 2014) | Hospital outpatient  Patient home  From Original paper (Batterham, 2014) | ''enrolled 8-16 weeks after discharge.''  From Original paper (Batterham, 2014) | 1 - 3 months  ''two hospital-based, physiotherapist-led supervised sessions per week. Participants were encouraged to add one unsupervised session each week of the same duration and intensity''  From Original paper (Batterham, 2014) |
| Zhang, 2023 | ICU follow-up services | Variable between sites | Variable between sites | Variable between sites | Variable between sites | Variable between sites | Variable between sites | Variable between sites |
| Zilahi 2019 | GP Information sharing / support | Information sharing between intensive care  and primary care after an episode of critical  illness | Unclear | General Practice teams associated with 200 GPs selected by proportionate stratified random sampling from the Irish  College of General Practitioners (ICGP) database which includes 85% of GPs in Ireland | ICU teams associated with Intensive care medicine consultants (ICMCs) in the Intensive Care Society of Ireland database  comprising >90% of ICMCs in Ireland | Information sharing from Intensive Care | Varied between participants | Varied between participants |

**Appendix 4 - Summary of study quality assessment**

|  | **Was there a clear statement of the aims of the research?** | **Is a qualitative methodology appropriate?** | **Was the research design appropriate to address the aims of the research?** | **Was the recruitment strategy appropriate to the aims of the research?** | **Was the data collected in a way that addressed the research issue?** | **Has the relationship between researcher and participants been adequately considered?** | **Have ethical issues been taken into consideration?** | **Was the data analysis sufficiently rigorous?** | **Is there a clear statement of findings?** | **Has the value of the research been considered?** |
| --- | --- | --- | --- | --- | --- | --- | --- | --- | --- | --- |
| **Backman 2018** | Yes | Yes | Yes | Yes | Yes | Cannot tell | Yes | Yes | Yes | Yes |
| **Bench 2016** | Yes | Yes | Cannot tell | Yes | Yes | Yes | Yes | Yes | Yes | Yes |
| **Boehm, 2023** | Yes | Yes | Yes | Yes | Yes | Yes | Yes | Yes | Yes | Yes |
| **Castro-Avila 2021** | Yes | Yes | Cannot tell | Yes | Yes | Yes | Yes | Yes | Yes | Yes |
| **Clarke, 2023** | Yes | Yes | Yes | Yes | Yes | Yes | Yes | Yes | Yes | Yes |
| **Clarke, 2024** | Yes | Yes | Yes | Yes | Yes | Yes | Yes | Yes | Yes | Yes |
| **Connolly 2014** | Yes | Cannot tell | Yes | Yes | Cannot tell | Cannot tell | Yes | Cannot tell | Cannot tell | Yes |
| **Connolly 2021** | Yes | Yes | Yes | Yes | Yes | Yes | No | Cannot tell | Yes | Yes |
| **Engstrom 2008** | Yes | Yes | Cannot tell | Yes | Yes | Cannot tell | Yes | Yes | Yes | Yes |
| **Engstrom 2010** | Yes | Yes | Cannot tell | Yes | Yes | Cannot tell | Yes | Yes | Yes | Yes |
| **Engstrom 2018** | Yes | Yes | Cannot tell | Cannot tell | Yes | Cannot tell | Yes | Yes | Yes | Yes |
| **Ferguson 2019** | Yes | Yes | Yes | Yes | Yes | Yes | Yes | Yes | Yes | Yes |
| **Gehrke-Beck 2021** | Yes | Yes | Yes | Yes | Yes | Yes | Yes | Yes | Yes | Yes |
| **Glaeemose, 2024** | Yes | Yes | Yes | Yes | Yes | Cannot tell | Yes | Yes | Yes | Yes |
| **Griffiths 2006** | Yes | Cannot tell | Cannot tell | Yes | Cannot tell | Cannot tell | Cannot tell | Cannot tell | Yes | Yes |
| **Haines 2019** | Yes | Yes | Yes | Yes | Yes | Yes | Yes | Yes | Yes | Yes |
| **Hanifa 2018** | Yes | Yes | Yes | Yes | Yes | Yes | Yes | Yes | Yes | Yes |
| **Hauschildt, 2022** | Yes | Yes | Yes | Yes | Yes | Cannot tell | Yes | Yes | Yes | Yes |
| **Henderson, 2022** | Yes | Cannot tell | Cannot tell | Cannot tell | Cannot tell | Cannot tell | Cannot tell | Cannot tell | Yes | Yes |
| **Jensen 2019** | Yes | Yes | Yes | Yes | Yes | Yes | Yes | Yes | Yes | Yes |
| **Kovaleva, 2023** | Yes | Yes | Yes | Yes | Yes | Yes | Yes | Yes | Yes | Yes |
| **Leggett, 2023** | Yes | Yes | Yes | Yes | Yes | Yes | Yes | Yes | Yes | Yes |
| **Leggett, 2024** | Yes | Yes | Yes | Yes | Yes | Yes | Yes | Yes | Yes | Yes |
| **Major 2021** | Yes | Yes | Yes | Cannot tell | Cannot tell | Cannot tell | Yes | Yes | Yes | Yes |
| **McPeake 2017** | Yes | Yes | Yes | Yes | Yes | Yes | Yes | Yes | Yes | Yes |
| **McPeake 2020** | Yes | Yes | Yes | Yes | Yes | Yes | Yes | Yes | Yes | Yes |
| **Parker 2020** | Yes | Yes | Yes | Yes | Yes | Yes | Yes | Yes | Yes | Yes |
| **Pattison 2007** | Yes | Yes | Yes | Yes | Yes | Cannot tell | Yes | Yes | Yes | Yes |
| **Petersson 2011** | Yes | Yes | Cannot tell | Yes | Yes | Cannot tell | Yes | Cannot tell | Yes | Yes |
| **Prevedello 2021** | Yes | Yes | Yes | Yes | Yes | Yes | Yes | Yes | Yes | Yes |
| **Prinjha 2009** | Yes | Yes | Yes | Yes | Yes | Yes | Yes | Yes | Yes | Yes |
| **Rohr 2021** | Yes | Yes | Yes | Yes | Yes | Cannot tell | Yes | Yes | Yes | Yes |
| **Sanftenberg, 2023** | Yes | Yes | Yes | Yes | Yes | Yes | Yes | Yes | Yes | Yes |
| **vanBeusekom 2018** | Yes | Yes | Yes | Cannot tell | Yes | Cannot tell | Yes | Yes | Yes | Yes |
| **Walker 2015** | Yes | Yes | Yes | Yes | Yes | Yes | Yes | Yes | Yes | Yes |
| **Zhang, 2023** | Yes | Yes | Yes | Yes | Yes | Yes | Yes | Yes | Yes | Yes |
| **Zilahi 2019** | Yes | Yes | Yes | Yes | Yes | Cannot tell | Yes | Yes | Yes | Yes |

**Appendix 5 – GRADE CERQual assessment of confidence in the review findings summary**

| **OVERARCHING PRINCIPALS** | | | | | | | |
| --- | --- | --- | --- | --- | --- | --- | --- |
| **Domain** | **Articles contributing data** | ***Methodological limitations*** | ***Coherence***  (Contradictory data, incomplete data or competing theories) | ***Adequacy***  (Richness and quantity of data) | ***Relevance***  (Data from primary studies is applicable  to the context specified in the review question) | **CERQual assessment of confidence** | **Explanation of CERQual assessment** |
| ***Person centred holistic care:***  Intervention should address, not just identify, unmet patient needs and gaps in care. | (Haines et al., 2019, Jensen et al., 2019, Glimelius Petersson et al., 2011, Boehm et al., 2023, Clarke et al., 2024) | Minor concerns  Petersson 2011  - Research design  - Participant-researcher relationship  - Data analysis | No, or very minor, concerns | No, or very minor, concerns | No, or very minor, concerns | High confidence | Minor or very minor concerns about methodological limitations, coherence, adequacy, and relevance. |
| ***Person centred holistic care:***  Staff should aim to build rapport and trust with patients through effective communication. | (Haines et al., 2019, Jensen et al., 2019, Glimelius Petersson et al., 2011, Hanifa et al., 2018, Ferguson et al., 2019, Pattison et al., 2007, Sanftenberg et al., 2023) | Minor concerns  Petersson 2011  - Research design  - Participant-researcher relationship  - Data analysis. | No, or very minor, concerns | No, or very minor, concerns | No, or very minor, concerns | High confidence | Minor or very minor concerns about methodological limitations, coherence, adequacy, and relevance. |
| ***Adaptable & personalised:***  Intervention should be adaptable to each patient’s specific unmet needs & recovery trajectory. | (Ferguson et al., 2019, Walker et al., 2015, McPeake et al., 2019, Bench et al., 2016, Prinjha et al., 2009, Henderson et al., 2022, Boehm et al., 2023, Clarke et al., 2024, Glæemose et al., 2024, Kovaleva et al., 2023, Zhang et al., 2023) | Minor concerns  Henderson 2022  - Insufficient information for multiple methodological domains  Bench 2016  - Research design  Glæemose 2024  - Participant-researcher relationship | No, or very minor, concerns | No, or very minor, concerns | No, or very minor, concerns | High confidence | Minor or very minor concerns about methodological limitations, coherence, adequacy, and relevance. |
| **Domain** | **Articles contributing data** | ***Methodological limitations*** | ***Coherence*** | ***Adequacy*** | ***Relevance*** | **CERQual assessment of confidence** | **Explanation of CERQual assessment** |
| **Adaptable & personalised:**  Intervention should be adaptable to the capability and capacity of each patient to engage (e.g. functional status, comorbidities and health literacy) | (Pattison et al., 2007, Glimelius Petersson et al., 2011, Gehrke-Beck et al., 2021, Ferguson et al., 2019, Prinjha et al., 2009, Parker et al., 2020, van Beusekom et al., 2018, Haines et al., 2019, Major et al., 2021) | Moderate concerns  Petersson 2011  - Research design  - Participant-researcher relationship  - Data analysis  Pattison 2007  - Participant-researcher relationship  Major 2021  - Recruitment strategy  - Data collection  - Participant-researcher relationship  van Beusekom 2018  - Recruitment strategy  - Participant-researcher relationship | No, or very minor, concerns | No, or very minor, concerns | No, or very minor, concerns | Moderate confidence | Moderate concerns about methodological limitations.  Minor or very minor concerns about coherence, adequacy, and relevance. |
| ***Adaptable & personalised****:*  Intervention should account for variable patient motivation to engage and beliefs about the intervention. | (Haines et al., 2019, Hanifa et al., 2018, Glimelius Petersson et al., 2011, Ferguson et al., 2019, van Beusekom et al., 2018, Glæemose et al., 2024, Zhang et al., 2023) | Minor concerns  Petersson 2011  - Research design  - Participant-researcher relationship  - Data analysis  van Beusekom 2018  - Recruitment strategy  - Participant-researcher relationship  Glæemose 2024  - Participant-researcher relationship | No, or very minor, concerns | No, or very minor, concerns | No, or very minor, concerns | High confidence | Minor or very minor concerns about methodological limitations, coherence, adequacy, and relevance. |
| ***Practicality:***  Intervention designers should consider the practicality of the intervention for staff and patients. | (Major et al., 2021, van Beusekom et al., 2018, Clarke et al., 2024) | Moderate concerns  Major 2021  - Recruitment strategy  - Data collection  - Participant-researcher relationship  Van Beusekom 2018  - Recruitment strategy  - Participant-researcher relationship | Minor concerns | Minor concerns | No, or very minor, concerns | Moderate confidence | Moderate concerns about methodological limitations. Minor concerns about adequacy. |
| **Domain** | **Articles contributing data** | ***Methodological limitations*** | ***Coherence*** | ***Adequacy*** | ***Relevance*** | **CERQual assessment of confidence** | **Explanation of CERQual assessment** |
| ***Continuity and coordination of care:***  Interventions should incorporate continuity and coordination of care to address fragmented care and lack of clarity on remit and responsibility. | (McPeake et al., 2020, Prinjha et al., 2009, Rohr et al., 2021, Prevedello et al., 2021, Castro-Avila et al., 2021, Boehm et al., 2023, Leggett et al., 2024, Sanftenberg et al., 2023). | Minor concerns  Rohr 2021  - Participant-researcher relationship  Castro-Avila. 2021  - Research design | No, or very minor, concerns | No, or very minor, concerns | No, or very minor, concerns | High confidence | Minor or very minor concerns about methodological limitations, coherence, adequacy and relevance. |
| ***Goal setting, normalising recovery, & validation of progress***  Staff should set achievable goals for patients and validates progress. | (Pattison et al., 2007, McPeake et al., 2020, Prinjha et al., 2009, Walker et al., 2015, Glæemose et al., 2024, Kovaleva et al., 2023, Leggett et al., 2024) | Minor concerns  Pattison 2007  - Participant-researcher relationship  Glæemose 2024  - Participant-researcher relationship | No, or very minor, concerns | No, or very minor, concerns | No, or very minor, concerns | High confidence | Minor or very minor concerns about methodological limitations, coherence, adequacy and relevance. |
| ***Involvement of informal carers***  Informal carers play important roles supporting patients in the post-hospital period, and their role should be considered in any intervention | (Haines et al., 2019, Hanifa et al., 2018, McPeake et al., 2017, Bench et al., 2016, Henderson et al., 2022, Glæemose et al., 2024, Kovaleva et al., 2023, Leggett et al., 2023) | Moderate concerns  Bench 2016  - Research design  Henderson 2022  - Insufficient information for multiple methodological domains  Glæemose 2024  - Participant-researcher relationship | No, or very minor, concerns | No, or very minor, concerns | No, or very minor, concerns | Moderate confidence | Minor or very minor concerns about coherence, adequacy, and relevance. Moderate concerns about methodological limitations. |

| **Potential components** | | | | | | | |
| --- | --- | --- | --- | --- | --- | --- | --- |
| **Domain** | **Articles contributing data** | ***Methodological limitations*** | ***Coherence*** | ***Adequacy*** | ***Relevance*** | **CERQual assessment of confidence** | **Explanation of CERQual assessment** |
| ***Physical Rehabilitation*** | | | | | | | |
| ***Patient needs and trajectory*:**  The choice of exercise should be accessible and adaptable to account for variable patient needs. | (Ferguson et al., 2019, Walker et al., 2015, Major et al., 2021) | Minor concerns  Major 2021  - Recruitment strategy  - Data collection  - Participant-researcher relationship | No, or very minor, concerns | Minor concerns  Data from 3 studies | No, or very minor, concerns | High confidence | Minor or very minor concerns about methodological limitations, coherence, adequacy, and relevance. |
| ***Patient motivation and beliefs***  Supervision of the physical rehabilitation programmes may enhance engagement and benefit. | (Ferguson et al., 2019, Walker et al., 2015) | No concerns | No, or very minor, concerns | Moderate concerns  Data from 2 studies | No, or very minor, concerns | Moderate confidence | Moderate concerns about adequacy. Very minor or no concerns about methodological limitations, coherence, and relevance. |
| ***Integration with existing outpatient & community services***  Designers need to consider how the intervention will integrate with existing outpatient and community physiotherapy services. | (van Beusekom et al., 2018, Walker et al., 2015, Zilahi and O’Connor, 2019, Connolly et al., 2014, Haines et al., 2019, Major et al., 2021, Castro-Avila et al., 2021) | Minor concerns  Major 2021  - Recruitment strategy  - Data collection  - Participant-researcher relationship  van Beusekom 2018  - Recruitment strategy  - Participant-researcher relationship  Zilahi 2019  - Participant-researcher relationship  Connolly 2014  - Insufficient information for multiple methodological domains  Castro-Avila. 2021  - Research design | No, or very minor, concerns | No, or very minor, concerns | No, or very minor, concerns | High confidence | Moderate concerns about methodological limitations. Minor or very minor concerns about coherence, adequacy, and relevance. |
| **Domain** | **Articles contributing data** | ***Methodological limitations*** | ***Coherence*** | ***Adequacy*** | ***Relevance*** | **CERQual assessment of confidence** | **Explanation of CERQual assessment** |
| ***Psychological rehabilitation (Therapy/Psychology)*** | | | | | | | |
| **Person centred holistic care**  Psychological therapy needs to be integrated with other forms of support | (Clarke et al., 2024, Kovaleva et al., 2023, Sanftenberg et al., 2023, Rohr et al., 2021, Bench et al., 2016, Hanifa et al., 2018, Prinjha et al., 2009) | No concerns | No, or very minor, concerns | Minor concerns  Data from 3 studies | No, or very minor, concerns | High confidence | Minor concerns about adequacy. Very minor or no concerns about methodological limitations, coherence, and relevance. |
| ***Patient capability & capacity***  Patients will vary in terms of their capability and capacity to return to the admitting hospital for therapy (e.g., physical functional or triggering effect of hospital). | (Clarke et al., 2024, Kovaleva et al., 2023) | No concerns | No, or very minor, concerns | Moderate concerns  Data from 2 studies | No, or very minor, concerns | Moderate confidence | Moderate concerns about adequacy. Very minor or no concerns about methodological limitations, coherence, and relevance. |
| ***Psychological rehabilitation (Information provision)*** | | | | | | | |
| **Patient needs and trajectory**  Personalised information to support patients make sense of their ICU stay and recovery should be offered provided via multiple sources to meet each patient’s individual needs. | (Rohr et al., 2021, Bench et al., 2016, Hanifa et al., 2018, Prinjha et al., 2009, Clarke et al., 2024). | Minor concerns  Rohr 2021  - Participant-researcher relationship  Bench 2016  - Research design | No, or very minor, concerns | No, or very minor, concerns | No, or very minor, concerns | High confidence | Minor or very minor concerns about methodological limitations, coherence, adequacy, and relevance. |
| ***Psychological rehabilitation (ICU visit)*** | | | | | | | |
| ***Patient capability & capacity****:*  Patients will vary in terms of their capability and capacity to return to the admitting hospital (e.g., functional status or geographic location). | (Engström et al., 2018, Engström et al., 2008, Prinjha et al., 2009) | Minor concerns  Engström 2018  - Research design  - Recruitment strategy  - Participant-research relationship  Engström 2008  - Research design  - Participant-research relationship | No, or very minor, concerns | Moderate concerns  Data from 3 studies, two of which are from the same research group. | No, or very minor, concerns | Moderate confidence | Minor or very minor concerns about methodological limitations, coherence, and relevance. Moderate concerns about adequacy. |
| **Domain** | **Articles contributing data** | ***Methodological limitations*** | ***Coherence*** | ***Adequacy*** | ***Relevance*** | **CERQual assessment of confidence** | **Explanation of CERQual assessment** |
| ***Practical considerations***  Adjuncts (e.g. ICU diary or photographs) may enhance benefit gained. | (Engström et al., 2008, Engström et al., 2018, Engström and Söderberg, 2010, Hanifa et al., 2018, Jensen et al., 2019) | Minor concerns  Engström 2008  - Research design  - Participant-research relationship  Engström 2010  - Research design  - Participant-research relationship  Engström 2018  - Research design  - Recruitment strategy  - Participant-research relationship | No, or very minor, concerns | No, or very minor, concerns | No, or very minor, concerns | High confidence | Minor or very minor concerns about methodological limitations, coherence, adequacy, and relevance. |
| ***Informal carer involvement***  Intervention designers need to consider whether to invite informal carers. | (Engström et al., 2008, Engström et al., 2018) | Minor concerns  Engström 2008  - Research design  - Participant-research relationship  Engström 2018  - Research design  - Recruitment strategy  - Participant-research relationship | No, or very minor, concerns | Moderate concerns  Data from 2 studies | No, or very minor, concerns | Moderate confidence | Minor or very minor concerns about methodological limitations, coherence, and relevance. Moderate concerns about adequacy. |
| ***Psychological rehabilitation (peer support)*** | | | | | | | |
| ***Patient needs and trajectory***  Optimal patient selection is required to ensure peer support is offered to patients who are most likely to benefit. | (Haines et al., 2019, McPeake et al., 2019, Kovaleva et al., 2023). | No concerns | Minor concerns | Minor concerns  Data from 3 studies | Minor concerns | High confidence | Minor or very minor concerns about methodological limitations, coherence, relevance, about adequacy. |
| ***Practical considerations***  Supervision, choice of model and structure for discussion | (Haines et al., 2019, McPeake et al., 2019, Glæemose et al., 2024). | Minor concerns  Glæemose 2024  - Participant-researcher relationship | Minor concerns | Minor concerns  Data from 3 studies | Minor concerns | High confidence | Minor concerns about methodological limitations, coherence, relevance and adequacy. |
| ***Informal carer involvement***  Intervention designers need to consider whether to invite informal carers | (Haines et al., 2019, Bäckman et al., 2018) | Minor concerns  Bäckman 2018  - Participant- researcher relationship | Minor concerns | Moderate concerns  Data from 2 studies | Minor concerns | Moderate confidence | Minor or very minor concerns about methodological limitations, coherence, and relevance. Moderate concerns about adequacy. |
| ***Medical review (Including medicines optimisation)*** | | | | | | | |
| ***Clarity on purpose & remit***  Staff engagement is dependent on their clarify on the purpose of incorporating medical review (e.g., comprehensive screening for unmet needs, follow-up of tests and referrals, or medication review) | (McPeake et al., 2020, Prinjha et al., 2009, Boehm et al., 2023) | No concerns | Minor concerns | Minor concerns | Minor concerns | High confidence | Minor or very minor concerns about methodological limitations, coherence, and relevance, and adequacy. |
| ***Staff capability***  Incorporating medical review will be dependent upon access to staff with the required experience. | (Connolly et al., 2014, Boehm et al., 2023, Kovaleva et al., 2023, Sanftenberg et al., 2023) | High concerns  Connolly 2014  - Insufficient information for multiple methodological domains | Minor concerns | Minor concerns | Minor concerns | High confidence | Minor or very minor concerns about methodological limitations, coherence, and relevance, and adequacy. |
| ***Socio-economic support*** | | | | | | | |
| ***Available Resources:***  Access to staff with the required expertise | (McPeake et al., 2020, Henderson et al., 2022, Boehm et al., 2023) | Moderate concerns  Henderson 2022  - Insufficient information for multiple methodological domains | Minor concerns | Minor concerns | Minor concerns | Moderate confidence | Moderate concerns about methodological limitations. Minor concerns about coherence, and relevance, and adequacy. |
| ***Integration with existing outpatient & community services****:*  Healthcare, social care and community services | (McPeake et al., 2020, Henderson et al., 2022, Boehm et al., 2023) | Moderate concerns  Henderson 2022  - Insufficient information for multiple methodological domains | Minor concerns | Minor concerns | Minor concerns | Moderate confidence | Moderate concerns about methodological limitations. Minor concerns about coherence, and relevance, and adequacy. |
| **Domain** | **Articles contributing data** | ***Methodological limitations*** | ***Coherence*** | ***Adequacy*** | ***Relevance*** | **CERQual assessment of confidence** | **Explanation of CERQual assessment** |
| ***Information sharing with primary care*** | | | | | | | |
| ***Integration with primary care***  Information sharing between secondary and primary care requires integration. | (Major et al., 2021, Bench et al., 2016, Castro-Avila et al., 2021, Connolly et al., 2021, Rohr et al., 2021, Leggett et al., 2023, Leggett et al., 2024) | Minor concerns  Major 2021  - Recruitment strategy  - Data collection  - Participant-researcher relationship  Bench 2016  - Research design  Castro-Avila. 2021  - Research design  Rohr 2021  - Participant-researcher relationship  Connolly 2021  - Data analysis | No, or very minor, concerns | No, or very minor, concerns | No, or very minor, concerns | High confidence | Minor or very minor concerns about methodological limitations, coherence, adequacy, and relevance. |
| ***Staff capability (Secondary Care):***  The quality of information provided by secondary care teams is dependent on the experience of the secondary care team member preparing it. | (Bench et al., 2016, Zilahi and O’Connor, 2019, Leggett et al., 2023, Leggett et al., 2024) | Minor concerns  Bench 2016  - Research design  Zilahi 2019  - Participant-researcher relationship | No, or very minor, concerns | Minor | No, or very minor, concerns | High confidence | Minor or very minor concerns about methodological limitations, coherence, relevance, and adequacy. |
| ***Staff capability (Primary Care):***  The ability of primary care teams to act on information received depends on their access to high quality and timely information | (Bench et al., 2016, Castro-Avila et al., 2021, Gehrke-Beck et al., 2021, Zilahi and O’Connor, 2019, Hauschildt et al., 2022, Leggett et al., 2023, Leggett et al., 2024) | Minor concerns  Zilahi 2019  - Participant-researcher relationship  Castro-Avila. 2021  - Research design  Bench 2016  - Research design  Hauschildt 2022  - Participant-researcher relationship | No, or very minor, concerns | No, or very minor, concerns | No, or very minor, concerns | High confidence | Minor or very minor concerns about methodological limitations, coherence, adequacy, and relevance. |
| **Domain** | **Articles contributing data** | ***Methodological limitations*** | ***Coherence*** | ***Adequacy*** | ***Relevance*** | **CERQual assessment of confidence** | **Explanation of CERQual assessment** |
| ***Staff capability (Primary Care):***  The ability of primary care staff to act on information received depends on their experience of ICU sequalae. | (Bench et al., 2016, Gehrke-Beck et al., 2021, Rohr et al., 2021, Prinjha et al., 2009, Leggett et al., 2024), | Minor concerns  Bench 2016  - Research design  Rohr 2021  - Participant-researcher relationship | No, or very minor, concerns | Minor concerns | No, or very minor, concerns | High confidence | Minor or very minor concerns about methodological limitations, coherence, adequacy, and relevance. |
| ***Staff capacity (Primary Care):***  The capacity of primary care staff to act on information received is impacted by factors including other workload pressures. | (Gehrke-Beck et al., 2021, Bench et al., 2016, Leggett et al., 2024). | Minor concerns  Bench 2016  - Research design | No, or very minor, concerns | Minor concerns | No, or very minor, concerns | High confidence | Minor or very minor concerns about methodological limitations, coherence, relevance and adequacy. |
| ***Staff motivation & beliefs (Primary Care):***  The motivation of primary care staff to act on information received depends on their understanding of their remit and responsibility. | (Bench et al., 2016, Castro-Avila et al., 2021, Zilahi and O’Connor, 2019, Leggett et al., 2024) | Minor concerns  Zilahi 2019  - Participant-researcher relationship  Castro-Avila. 2021  - Research design  Bench 2016  - Research design | No, or very minor, concerns | Minor concerns | No, or very minor, concerns | High confidence | Minor or very minor concerns about methodological limitations, coherence, adequacy, and relevance. |

| **Design considerations** | | | | | | | |
| --- | --- | --- | --- | --- | --- | --- | --- |
| **Domain** | **Articles contributing data** | ***Methodological limitations*** | ***Coherence*** | ***Adequacy*** | ***Relevance*** | **CERQual assessment of confidence** | **Explanation of CERQual assessment** |
| ***Who (patients)*** | | | | | | | |
| ***Optimal patient selection****:*  Interventions should target patients who are most likely to benefit. | (Haines et al., 2019, Rohr et al., 2021, Major et al., 2021) | Minor concerns  Major 2021  - Recruitment strategy  - Data collection  - Participant-researcher relationship  Rohr 2021  - Participant-researcher relationship | Minor concerns | Minor concerns  Inferred from data from 3 studies | Minor concerns | High confidence | Minor or very minor concerns about methodological limitations, coherence, adequacy, and relevance. |
| ***Who (Staff): MDT vs Care coordinator*** | | | | | | | |
| ***Staff capability:***  Successful implementation of any intervention requires access to staff with the required skills and experience. | (Gehrke-Beck et al., 2021, Hanifa et al., 2018, Jensen et al., 2019, Pattison et al., 2007, Glimelius Petersson et al., 2011, Ferguson et al., 2019, Connolly et al., 2014, Haines et al., 2019, Major et al., 2021, Rohr et al., 2021, Clarke et al., 2023, Kovaleva et al., 2023, Leggett et al., 2023, Sanftenberg et al., 2023, Zhang et al., 2023). | Moderate concerns  Petersson 2011  - Research design  - Participant-researcher relationship  - Data analysis  Pattison 2007  - Participant-researcher relationship  Major 2021  - Recruitment strategy  - Data collection  - Participant-researcher relationship  Rohr 2021  - Participant-researcher relationship  Connolly 2014  - Multiple areas | No, or very minor, concerns | No, or very minor, concerns | No, or very minor, concerns | Moderate confidence | Minor or very minor concerns about coherence, adequacy, and relevance. Moderate concerns about methodological limitations. |
| **Domain** | **Articles contributing data** | ***Methodological limitations*** | ***Coherence*** | ***Adequacy*** | ***Relevance*** | **CERQual assessment of confidence** | **Explanation of CERQual assessment** |
| ***Resources*:**  Successful implementation of an intervention requires access to the required resources (E.g. staffing and funding) | (Haines et al., 2019, Connolly et al., 2021, Major et al., 2021, Prevedello et al., 2021, Rohr et al., 2021, Henderson et al., 2022, Connolly et al., 2014, Castro-Avila et al., 2021, Griffiths et al., 2006, van Beusekom et al., 2018, Leggett et al., 2024) | Moderate concerns  Henderson 2022  - Multiple areas  Major 2021  - Recruitment strategy  - Data collection  - Participant-researcher relationship  Rohr 2021  - Participant-researcher relationship  Connolly 2014  - Multiple areas  Castro-Avila. 2021  - Research design | No, or very minor, concerns | No concerns | No, or very minor, concerns | Moderate confidence | Minor or very minor concerns about methodological limitations, coherence, adequacy, and relevance. |
| ***Integration with existing services***  Intervention designers needs to consider if and how the intervention will integrate with staff from existing outpatient and community services | (van Beusekom et al., 2018, Walker et al., 2015, Zilahi and O’Connor, 2019, Connolly et al., 2014, Haines et al., 2019, Major et al., 2021, Castro-Avila et al., 2021, Prinjha et al., 2009, Griffiths et al., 2006, Rohr et al., 2021, Bench et al., 2016) | Minor concerns  Major 2021  - Recruitment strategy  - Data collection  - Participant-researcher relationship  Van Beusekom 2018  - Recruitment strategy  - Participant-researcher relationship  Zilahi 2019  - Participant-researcher relationship  Connolly 2014  - Multiple areas  Castro-Avila. 2021  - Research design | No, or very minor, concerns | No concerns | No, or very minor, concerns | High confidence | Minor or very minor concerns about methodological limitations, coherence, adequacy, and relevance. |
| ***Staff motivation & beliefs***  ***Culture: Remit & responsibility***  For successful implementation, staff need to believe provision of ICU follow-up is important and falls within their remit and responsibility. | (Bench et al., 2016, Castro-Avila et al., 2021, Leggett et al., 2024, Zhang et al., 2023) | Minor concerns  Bench 2016  - Research design  Castro-Avila. 2021  - Research design | No, or very minor, concerns | Minor concerns | No, or very minor, concerns | High confidence | Minor or very minor concerns about methodological limitations, coherence, relevance, and adequacy. |
| **Domain** | **Articles contributing data** | ***Methodological limitations*** | ***Coherence*** | ***Adequacy*** | ***Relevance*** | **CERQual assessment of confidence** | **Explanation of CERQual assessment** |
| ***Where (Home vs outpatient clinic)*** | | | | | | | |
| ***Staff capability:***  The location of intervention implementation will impact access to multidisciplinary experience to address multidimensional patient needs | (Haines et al., 2019, Connolly et al., 2021, Griffiths et al., 2006, Henderson et al., 2022, McPeake et al., 2017, McPeake et al., 2020, Pattison et al., 2007, Prevedello et al., 2021, Prinjha et al., 2009, Hanifa et al., 2018, Major et al., 2021, Sanftenberg et al., 2023). | Moderate concerns  Henderson 2022  - Multiple areas  Pattison 2007  - Participant-researcher relationship  Connolly 2021  - Data analysis  Griffiths 2006  - Multiple areas  Major 2021  - Recruitment strategy  - Data collection  - Participant-researcher relationship | No, or very minor, concerns | No concerns | No, or very minor, concerns | Moderate confidence | Minor or very minor concerns about adequacy, coherence, and relevance. Moderate concerns about methodological limitations. |
| ***Patient capacity and capacity:***  The location of intervention implementation will impact the capability and capacity of patients to engage  (e.g., physical, psychological and cognitive function, triggering effect of the hospital, work / family commitments and access to transport). | (Major et al., 2021, Glimelius Petersson et al., 2011, Gehrke-Beck et al., 2021, Haines et al., 2019, Clarke et al., 2023, Glæemose et al., 2024, Kovaleva et al., 2023, Sanftenberg et al., 2023, Zhang et al., 2023). | Moderate concerns  Petersson 2011  - Research design  - Participant-researcher relationship  - Data analysis  Major 2021  - Recruitment strategy  - Data collection  - Participant-researcher relationship  Glæemose 2024  - Participant-researcher relationship | No, or very minor, concerns | No, or very minor, concerns | No, or very minor, concerns | Moderate confidence | Minor or very minor concerns about adequacy, coherence, and relevance. Moderate concerns about methodological limitations. |
| **Domain** | **Articles contributing data** | ***Methodological limitations*** | ***Coherence*** | ***Adequacy*** | ***Relevance*** | **CERQual assessment of confidence** | **Explanation of CERQual assessment** |
| ***How (In-person vs virtual)*** | | | | | | | |
| ***Staff beliefs:***  Staff belief about the impact of virtual care will impact their motivation to engage. | (Parker et al., 2020, van Beusekom et al., 2018, Jensen et al., 2019, Zhang et al., 2023) | Minor concerns  van Beusekom 2018  - Recruitment strategy  - Participant-researcher relationship | Minor concerns | Minor concerns | No, or very minor, concerns | Moderate confidence | Minor or very minor concerns about methodological limitations, adequacy, coherence, and relevance. |
| ***Practical considerations***  Certain intervention components will require significant adaption to be delivered virtually. | (Major et al., 2021, Jensen et al., 2019, Kovaleva et al., 2023, Zhang et al., 2023) | Minor concerns  Major 2021  - Recruitment strategy  - Data collection  - Participant-researcher relationship | Minor concerns | Minor concerns | No, or very minor, concerns | High confidence | Minor or very minor concerns about methodological limitations, coherence, relevance and adequacy. |
| ***Patient capability & capacity****:*  ***Patient motivation & beliefs:***  Virtual care has the potential to impact patient access negatively and positively, depending on each patients’ specific capability, capacity and beliefs. | (Parker et al., 2020, van Beusekom et al., 2018, Clarke et al., 2023, Kovaleva et al., 2023, Zhang et al., 2023) | Minor concerns  van Beusekom 2018  - Recruitment strategy  - Participant-researcher relationship | Minor concerns | Minor concerns | No, or very minor, concerns | High confidence | Minor or very minor concerns about methodological limitations, coherence, relevance, and adequacy. |
| ***When (Early vs later)*** | | | | | | | |
| ***Compatibility***  The timing of an intervention will be dependent on compatibility with specific intervention components | (Hanifa et al., 2018, Henderson et al., 2022) | Moderate concerns  Henderson 2022  - Multiple areas | Minor concerns | Moderate concerns  Data from 2 studies | No, or very minor, concerns | Low confidence | Minor or very minor concerns about methodological limitations, coherence, and relevance. Moderate concerns about adequacy. |
| ***Adaptable & personalised: Patient needs & trajectory*:**  The timing of interventions needs to be adaptable and account for the variable needs and recovery trajectory of patients. | (Hanifa et al., 2018, Connolly et al., 2014, Prinjha et al., 2009, Walker et al., 2015, Jensen et al., 2019, Major et al., 2021, Rohr et al., 2021, Glimelius Petersson et al., 2011, Engström et al., 2018, Clarke et al., 2024, Glæemose et al., 2024) | Minor concerns  Petersson 2011  - Research design  - Participant-researcher relationship  - Data analysis  Major 2021  - Recruitment strategy  - Data collection  - Participant-researcher relationship  Connolly 2014  - Multiple areas  Glæemose 2024  - Participant-researcher relationship | No, or very minor, concerns | No, or very minor, concerns | No, or very minor, concerns | High confidence | Minor or very minor concerns about methodological limitations, coherence, adequacy, and relevance. |
| **Domain** | **Articles contributing data** | ***Methodological limitations*** | ***Coherence*** | ***Adequacy*** | ***Relevance*** | **CERQual assessment of confidence** | **Explanation of CERQual assessment** |
| ***How much (Short- vs long-term)*** | | | | | | | |
| ***Adaptable & personalised:***  ***Patient needs & trajectory:***  The duration of follow-up will likely need to be adaptable to account for variable patient recovery trajectories. | (Prinjha et al., 2009, Major et al., 2021, Glimelius Petersson et al., 2011, Hanifa et al., 2018, Castro-Avila et al., 2021, Clarke et al., 2023, Kovaleva et al., 2023, Sanftenberg et al., 2023). | Minor concerns  Petersson 2011  - Research design  - Participant-researcher relationship  - Data analysis  Major 2021  - Recruitment strategy  - Data collection  - Participant-researcher relationship  Castro-Avila. 2021  - Research design | No, or very minor, concerns | No, or very minor, concerns | No, or very minor, concerns | High confidence | Minor or very minor concerns about methodological limitations, coherence, adequacy, and relevance. |
| ***Patient motivation:***  The duration of follow-up needs to balancing supporting patients against development of dependency. | (McPeake et al., 2020, Rohr et al., 2021, Ferguson et al., 2019, McPeake et al., 2017, Clarke et al., 2023) | Minor concerns  Rohr 2021  - Participant-researcher relationship | No, or very minor, concerns | No, or very minor, concerns | No, or very minor, concerns | High confidence | Minor or very minor concerns about methodological limitations, coherence, adequacy, and relevance. |

**References**

BÄCKMAN, C. G., AHLBERG, M., JONES, C. & FRISMAN, G. H. 2018. Group meetings after critical illness—Giving and receiving strength. *Intensive and Critical Care Nursing,* 46**,** 86-91.

BENCH, S., CORNISH, J. & XYRICHIS, A. 2016. Intensive care discharge summaries for general practice staff: a focus group study. *British Journal of General Practice,* 66**,** e904-e912.

BOEHM, L. M., DANESH, V., EATON, T. L., MCPEAKE, J., PENA, M. A., BONNET, K. R., STOLLINGS, J. L., JONES, A. C., SCHLUNDT, D. G. & SEVIN, C. M. 2023. Multidisciplinary ICU Recovery Clinic Visits: A Qualitative Analysis of Patient-Provider Dialogues. *CHEST,* 163**,** 843-854.

CASTRO-AVILA, A. C., JEFFERSON, L., DALE, V. & BLOOR, K. 2021. Support and follow-up needs of patients discharged from intensive care after severe COVID-19: a mixed-methods study of the views of UK general practitioners and intensive care staff during the pandemic’s first wave. *BMJ Open,* 11**,** e048392-e048392.

CLARKE, R., CHOW, H. & KERRISON, K. 2023. An Intensive Care Unit peer support group: Participants’ views on format, content and the impact on recovery journeys. *Journal of the Intensive Care Society,* 24**,** 258-264.

CLARKE, R., WEARE, V., CHOW, H., BOWERING-SHEEHAN, L. & HITCHCOCK, C. 2024. “It saved me”: A thematic analysis of experiences of psychological therapy following critical illness and intensive care. *Journal of the Intensive Care Society*.

CONNOLLY, B., DOUIRI, A., STEIER, J., MOXHAM, J., DENEHY, L. & HART, N. 2014. A UK survey of rehabilitation following critical illness: Implementation of NICE Clinical Guidance 83 (CG83) following hospital discharge. *BMJ Open,* 4**,** 4963-4963.

CONNOLLY, B., MILTON-COLE, R., ADAMS, C., BATTLE, C., MCPEAKE, J., QUASIM, T., SILVERSIDES, J., SLACK, A., WALDMANN, C., WILSON, E. & MEYER, J. 2021. Recovery, rehabilitation and follow-up services following critical illness: an updated UK national cross-sectional survey and progress report. *BMJ Open,* 11**,** e052214.

ENGSTRÖM, Å., ANDERSSON, S. & SÖDERBERG, S. 2008. Re-visiting the ICU: Experiences of follow-up visits to an ICU after discharge: A qualitative study. *Intensive and Critical Care Nursing,* 24**,** 233-241.

ENGSTRÖM, Å., ROGMALM, K., MARKLUND, L. & WÄLIVAARA, B.-M. 2018. Follow-up visit in an ICU: receiving a sense of coherence. *Nursing in Critical Care,* 23**,** 308-315.

ENGSTRÖM, Å. & SÖDERBERG, S. 2010. Critical care nurses’ experiences of follow-up visits to an ICU. *Journal of Clinical Nursing,* 19**,** 2925-2932.

FERGUSON, K., BRADLEY, J. M., MCAULEY, D. F., BLACKWOOD, B. & O’NEILL, B. 2019. Patients’ Perceptions of an Exercise Program Delivered Following Discharge From Hospital After Critical Illness (the Revive Trial). *Journal of Intensive Care Medicine,* 34**,** 978-984.

GEHRKE-BECK, S., GENSICHEN, J., TURNER, K. M., HEINTZE, C. & SCHMIDT, K. F. 2021. General practitioners’ views and experiences in caring for patients after sepsis: a qualitative interview study. *BMJ Open,* 11**,** e040533.

GLÆEMOSE, A. O., HANIFA, A. L. B. & HASLUND-THOMSEN, H. 2024. Peer support in intensive care unit follow-up: A qualitative evaluation. *Nursing in Critical Care*.

GLIMELIUS PETERSSON, C., BERGBOM, I., BRODERSEN, K. & RINGDAL, M. 2011. Patients' participation in and evaluation of a follow-up program following intensive care. *Acta Anaesthesiologica Scandinavica,* 55**,** 827-834.

GRIFFITHS, J. A., BARBER, V. S., CUTHBERTSON, B. H. & YOUNG, J. D. 2006. A national survey of intensive care follow-up clinics. *Anaesthesia,* 61**,** 950-955.

HAINES, K. J., MCPEAKE, J., HIBBERT, E., BOEHM, L. M., APARANJI, K., BAKHRU, R. N., BASTIN, A. J., BEESLEY, S. J., BEVERIDGE, L., BUTCHER, B. W., DRUMRIGHT, K., EATON, T. L., FARLEY, T., FIRSHMAN, P., FRITSCHLE, A., HOLDSWORTH, C., HOPE, A. A., JOHNSON, A., KENES, M. T., KHAN, B. A., KLOOS, J. A., KROSS, E. K., MACTAVISH, P., MEYER, J., MONTGOMERY-YATES, A., QUASIM, T., SAFT, H. L., SLACK, A., STOLLINGS, J., WEINHOUSE, G., WHITTEN, J., NETZER, G., HOPKINS, R. O., MIKKELSEN, M. E., IWASHYNA, T. J. & SEVIN, C. M. 2019. Enablers and barriers to implementing ICU follow-up clinics and peer support groups following critical illness: The thrive collaboratives. *Critical Care Medicine,* 47**,** 1194-1200.

HANIFA, A. L. B., GLÆEMOSE, A. O. & LAURSEN, B. S. 2018. Picking up the pieces: Qualitative evaluation of follow-up consultations post intensive care admission. *Intensive and Critical Care Nursing,* 48**,** 85-91.

HAUSCHILDT, K. E., HECHTMAN, R. K., PRESCOTT, H. C. & IWASHYNA, T. J. 2022. Hospital Discharge Summaries Are Insufficient Following ICU Stays: A Qualitative Study. *Critical Care Explorations,* 4**,** e0715.

HENDERSON, P., QUASIM, T., SHAW, M., MACTAVISH, P., DEVINE, H., DANIEL, M., NICOLSON, F., O’BRIEN, P., WEIR, A., STRACHAN, L., SENIOR, L., LUCIE, P., BOLLAN, L., DUFFTY, J., HOGG, L., ROSS, C., SIM, M., SUNDARAM, R., IWASHYNA, T. J. & MCPEAKE, J. 2022. Evaluation of a health and social care programme to improve outcomes following critical illness: a multicentre study. *Thorax***,** thoraxjnl-2021-218428.

JENSEN, J. F., OVERGAARD, D., BESTLE, M. H., CHRISTENSEN, D. F., RATTRAY, J. & EGEROD, I. 2019. Intervention fidelity in postintensive care follow-up consultations at ten sites in the RAPIT-trial: A mixed-methods evaluation. *Journal of Advanced Nursing,* 75**,** 862-875.

KOVALEVA, M. A., JONES, A. C., KIMPEL, C. C., LAUDERDALE, J., SEVIN, C. M., STOLLINGS, J. L., JACKSON, J. C. & BOEHM, L. M. 2023. Patient and caregiver experiences with a telemedicine intensive care unit recovery clinic. *Heart & Lung: The Journal of Cardiopulmonary and Acute Care,* 58**,** 47-53.

LEGGETT, N., EMERY, K., ROLLINSON, T. C., DEANE, A., FRENCH, C., MANSKI NANKERVIS, J.-A., EASTWOOD, G., MILES, B., MEROLLI, M., ALI ABDELHAMID, Y. & HAINES, K. J. 2023. Fragmentation of care between intensive and primary care settings and opportunities for improvement. *Thorax,* 78**,** 1181-1187.

LEGGETT, N., EMERY, K., ROLLINSON, T. C., DEANE, A. M., FRENCH, C., MANSKI-NANKERVIS, J.-A., EASTWOOD, G., MILES, B., WITHERSPOON, S., STEWART, J., MEROLLI, M., ALI ABDELHAMID, Y. & HAINES, K. J. 2024. Clinician- and Patient-Identified Solutions to Reduce the Fragmentation of Post-ICU Care in Australia. *CHEST*.

MAJOR, M. E., DETTLING-IHNENFELDT, D., RAMAEKERS, S. P. J., ENGELBERT, R. H. H. & VAN DER SCHAAF, M. 2021. Feasibility of a home-based interdisciplinary rehabilitation program for patients with Post-Intensive Care Syndrome: the REACH study. *Critical Care,* 25**,** 279.

MCPEAKE, J., BOEHM, L. M., HIBBERT, E., BAKHRU, R. N., BASTIN, A. J., BUTCHER, B. W., EATON, T. L., HARRIS, W., HOPE, A. A., JACKSON, J., JOHNSON, A., KLOOS, J. A., KORZICK, K. A., MACTAVISH, P., MEYER, J., MONTGOMERY-YATES, A., QUASIM, T., SLACK, A., WADE, D., STILL, M., NETZER, G., HOPKINS, R. O., MIKKELSEN, M. E., IWASHYNA, T. J., HAINES, K. J. & SEVIN, C. M. 2020. Key Components of ICU Recovery Programs: What Did Patients Report Provided Benefit? *Critical Care Explorations,* 2**,** e0088-e0088.

MCPEAKE, J., HIRSHBERG, E. L., CHRISTIE, L. M., DRUMRIGHT, K., HAINES, K., HOUGH, C. L., MEYER, J., WADE, D., ANDREWS, A., BAKHRU, R., BATES, S., BARWISE, J. A., BASTARACHE, J., BEESLEY, S. J., BOEHM, L. M., BROWN, S., CLAY, A. S., FIRSHMAN, P., GREENBERG, S., HARRIS, W., HILL, C., HODGSON, C., HOLDSWORTH, C., HOPE, A. A., HOPKINS, R. O., HOWELL, D. C. J., JANSSEN, A., JACKSON, J. C., JOHNSON, A., KROSS, E. K., LAMAS, D., MACLEOD-SMITH, B., MANDEL, R., MARSHALL, J., MIKKELSEN, M. E., NACKINO, M., QUASIM, T., SEVIN, C. M., SLACK, A., SPURR, R., STILL, M., THOMPSON, C., WEINHOUSE, G., WILCOX, M. E. & IWASHYNA, T. J. 2019. Models of Peer Support to Remediate Post-Intensive Care Syndrome: A Report Developed by the Society of Critical Care Medicine Thrive International Peer Support Collaborative. *Critical Care Medicine,* 47**,** e21-e27.

MCPEAKE, J., SHAW, M., IWASHYNA, T. J., DANIEL, M., DEVINE, H., JARVIE, L., KINSELLA, J., MACTAVISH, P. & QUASIM, T. 2017. Intensive Care Syndrome: Promoting Independence and Return to Employment (InS:PIRE). Early evaluation of a complex intervention. *PLOS ONE,* 12**,** e0188028.

PARKER, A. M., NELLIOT, A., CHESSARE, C. M., MALIK, A. M., KONERU, M., HOSEY, M. M., OZOK, A. A., LYONS, K. D. & NEEDHAM, D. M. 2020. Usability and acceptability of a mobile application prototype for a combined behavioural activation and physical rehabilitation intervention in acute respiratory failure survivors. *Australian Critical Care,* 33**,** 511-517.

PATTISON, N. A., DOLAN, S., TOWNSEND, P. & TOWNSEND, R. 2007. After critical care: a study to explore patients’ experiences of a follow-up service. *Journal of Clinical Nursing,* 16**,** 2122-2131.

PREVEDELLO, D., STECKELMACHER, C., DEVROEY, M., NJIMI, H., CRETEUR, J. & PREISER, J.-C. 2021. The burden of implementation: A mixed methods study on barriers to an ICU follow-up program. *Journal of Critical Care,* 65**,** 170-176.

PRINJHA, S., FIELD, K. & ROWAN, K. 2009. What patients think about ICU follow-up services: a qualitative study. *Critical Care,* 13**,** R46.

ROHR, M., WEIß, A., BEIN, T., BERNARDI, C., BRANDSTETTER, S., DREWITZ, K. P., FISSER, C., LOSS, J., MALFERTHEINER, M. & APFELBACHER, C. 2021. Experiences, opinions and expectations of health care providers towards an intensive care unit follow-up clinic: Qualitative study and online survey. *Intensive and Critical Care Nursing,* 67**,** 103084.

SANFTENBERG, L., BEUTEL, A., FRIEMEL, C. M., KOSILEK, R. P., SCHAUER, M., ELBERT, T., REIPS, U.-D., GEHRKE-BECK, S., SCHUBERT, T., SCHMIDT, K., GENSICHEN, J., ADRION, C., ANGSTWURM, M., BERGMANN, A., BIELMEIER, G., BISCHHOFF, A., BOGDANSKI, R., BRETTNER, F., BRETTSCHNEIDER, C., BRIEGEL, J., BÜRKLE, M., DOHMANN, J., FALKAI, P., FELBINGER, T., FISCH, R., FÖRSTL, H., FOHR, B., FRANZ, M., FRIEDERICH, P., FRIEMEL, C. M., GALLINAT, J., GERLACH, H., GÜLDNER, A., HARDT, H., HEINTZE, C., HEINZ, A., HELLER, A., VON HEYMANN, C., HOPPMANN, P., HUGE, V., IRLBECK, M., JASCHINSKI, U., JARCZAK, D., JOOS, S., KAISER, E., KERINN, M., KLEFISCH, F.-R., KLUGE, S., KOCH, R., KOCH, T., KOWALSKI, M., KÖNIG, H.-H., LACKERMEIER, P., LAUGWITZ, K.-L., LEMKE, Y., LIES, A., LINDE, K., LINDEMANN, D., LÜHMANN, D., MAY, S., NEY, L., OLTROGGE, J., PANKOW, W., PAPIOL, S., RAGALLER, M., RANK, N., REILL, L., RICHTER, H.-P., RIESSEN, R., RINGEIS, G., RÜCHHARDT, A., SCHELLING, G., SCHELLING, J., SCHERAG, A., SCHERER, M., SCHNEIDER, A., SCHNEIDER, G., SCHNEIDER, J., SCHNURR, J., SCHULTZ, S., SCHULZE, T. G., SCHUMACHER, K., SPIETH, P., THURM, F., VOGL, T., VOIGT, K., WALTHER, A., WASSILOWSKY, D., WÄSCHER, C., WEBER-CARSTENS, S., WEHRSTEDT, R., WEIERSTALL-PUST, R., WEIS, M., WEISS, G., WELL, H., ZÖLLNER, C., ZWISSLER, B. & FOR THE, P.-S. T. 2023. Barriers and opportunities for implementation of a brief psychological intervention for post-ICU mental distress in the primary care setting – results from a qualitative sub-study of the PICTURE trial. *BMC Primary Care,* 24**,** 113.

VAN BEUSEKOM, I., BAKHSHI-RAIEZ, F., DE KEIZER, N. F., DONGELMANS, D. A. & VAN DER SCHAAF, M. 2018. Lessons learnt during the implementation of a web-based triage tool for Dutch intensive care follow-up clinics. *BMJ Open,* 8**,** e021249.

WALKER, W., WRIGHT, J., DANJOUX, G., HOWELL, S. J., MARTIN, D. & BONNER, S. 2015. Project Post Intensive Care eXercise (PIX): A qualitative exploration of intensive care unit survivors’ perceptions of quality of life post-discharge and experience of exercise rehabilitation. *Journal of the Intensive Care Society,* 16**,** 37-44.

ZHANG, F., CHEN, Z., XUE, D.-D., ZHANG, R. & CHENG, Y. 2023. Barriers and facilitators to offering post-intensive care follow-up services from the perspective of critical care professionals: A qualitative study. *Nursing in Critical Care*.

ZILAHI, G. & O’CONNOR, E. 2019. Information sharing between intensive care and primary care after an episode of critical illness; A mixed methods analysis. *PLOS ONE,* 14**,** e0212438-e0212438.
